# Rewiring Biological Barriers in Pancreatic Diseases with Metal–Organic Frameworks

**DOI:** 10.3390/biology15191783

**Published:** 2026-10-08

**Authors:** Hao Li, Jiewen Hou, Congzhen He, Changxing Liu, Bowen Ran, Xinzhe Song, Lanxiao Lin, Lan Bao, Yi Zhao, Jing Mu, Liwei Chen, Hui-Zi Huang

**Affiliations:** 1 College of Biological and Chemical Engineering, Qilu Institute of Technology, Jinan 250200, China; 2 Ministry of Education Key Laboratory of Cluster Science, Beijing Key Laboratory of Photoelectronic/Electrophotonic Conversion Materials, Analysis and Testing Center, Frontiers Science Center for High Energy Material, Advanced Technology Research Institute (Jinan), School of Chemistry and Chemical Engineering, Beijing Institute of Technology, Beijing 100081, China

**Keywords:** metal–organic frameworks, pancreatic cancer, drug delivery, tumor microenvironment, ferroptosis, cuproptosis, immunotherapy, theranostics

## Abstract

Pancreatic diseases, particularly pancreatic ductal adenocarcinoma (PDAC), remain among the most challenging disorders to diagnose and treat owing to a series of interconnected biological barriers, including dense stromal desmoplasia, profound immunosuppression, hypoxia, therapeutic resistance, and the lack of effective early-detection strategies. Overcoming these barriers requires multifunctional platforms capable of integrating diagnosis, microenvironment regulation, and precision therapy within a single system. Metal–organic frameworks (MOFs), a class of highly tunable porous crystalline materials, have emerged as uniquely suited candidates for this purpose because of their programmable composition, structural versatility, catalytic activity, and capacity for multifunctional integration. Notably, MOFs have evolved from passive drug carriers into active regulators of disease-associated biological processes, enabling enhanced drug delivery, extracellular matrix remodeling, ferroptosis- and cuproptosis-based precision therapy, energy-triggered reactive oxygen species generation, immune microenvironment reprogramming, and imaging-guided theranostics. Beyond pancreatic cancer, emerging evidence further supports their potential applications in pancreatitis management and interventional therapeutic enhancement. In this review, we systematically summarize recent advances in the rational engineering of MOFs for pancreatic diseases, focusing on how material design strategies are leveraged to overcome key pathological barriers and improve therapeutic outcomes. We further discuss current challenges associated with biosafety, biodegradation, large-scale manufacturing, and clinical translation, while highlighting future opportunities for the development of next-generation MOF-based precision nanomedicines. By integrating advances across materials science, nanomedicine, and pancreatic disease biology, this review provides a comprehensive framework for the design and translational development of MOF-enabled diagnostic and therapeutic platforms.

## Simple Summary

Pancreatic cancer remains highly challenging to treat because its dense and complex tumor environment restricts drug delivery and therapeutic efficacy. Metal–organic frameworks are versatile porous materials that can be rationally engineered to address these biological barriers. This review summarizes recent advances in their application to pancreatic diseases, particula rly pancreatic ductal adenocarcinoma, focusing on drug delivery, tumor microenvironment modulation, ferroptosis, cuproptosis, immunotherapy, and integrated diagnosis and treatment. We further highlight how precise control of framework structure, surface properties, and biological responsiveness enables multifunctional therapeutic strategies. Finally, current challenges in safety, delivery, biodegradation, manufacturing, and clinical translation are discussed. Overall, rationally engineered metal–organic frameworks represent a promising platform for overcoming the complex therapeutic barriers of pancreatic cancer and advancing precision nanomedicine.

## 1. Introduction

Pancreatic diseases, particularly pancreatic ductal adenocarcinoma (PDAC), remain among the most formidable challenges in contemporary medicine. Despite substantial advances in surgery, chemotherapy, radiotherapy, and targeted interventions, the clinical outcomes of pancreatic cancer have improved only marginally over recent decades [1,2,3,4,5]. The exceptionally poor prognosis of PDAC is largely attributed to a series of interconnected biological barriers, including extensive desmoplastic stroma, severe hypovascularization and hypoxia, profound immunosuppression, metabolic plasticity, and intrinsic therapeutic resistance [6,7,8]. These pathological features not only restrict drug accumulation and intratumoral penetration but also impair the efficacy of emerging therapeutic modalities such as reactive oxygen species (ROS)-based therapy, immunotherapy, and precision nanomedicine [9,10,11,12]. Consequently, overcoming the complex tumor microenvironment and achieving coordinated regulation of multiple pathological processes have become central challenges in the development of next-generation therapeutic strategies for pancreatic diseases [13,14,15,16,17].

Although PDAC represents the primary focus of current MOF-based pancreatic disease research owing to its high clinical burden and complex biological barriers, emerging studies have also highlighted the potential of MOF platforms in non-malignant pancreatic disorders, including pancreatitis and fibrosis-associated pancreatic injury. Therefore, this review primarily emphasizes PDAC while extending the discussion to broader pancreatic diseases where MOF-based strategies may provide unique therapeutic opportunities.

Among the diverse nanomaterial platforms investigated for pancreatic disease intervention, metal–organic frameworks (MOFs) have emerged as particularly promising candidates owing to their highly tunable composition, hierarchical porosity, large surface area, versatile surface chemistry, and intrinsic structural modularity [18,19,20,21,22]. Unlike conventional nanocarriers that primarily serve as passive drug reservoirs, MOFs provide an adaptable platform for integrating therapeutic, diagnostic, and catalytic functions within a single architecture [23,24,25,26,27]. Through rational manipulation of metal nodes, organic ligands, pore structures, and surface functionalities, MOFs can be engineered to regulate drug loading and release, remodel the tumor microenvironment, catalyze redox reactions, amplify therapeutic signals, activate antitumor immunity, and enable multimodal imaging [28,29,30,31]. These unique characteristics position MOFs at the intersection of materials science, nanomedicine, catalysis, and precision oncology, offering unprecedented opportunities to address the multifaceted challenges associated with pancreatic diseases [32,33,34,35,36].

Over the past decade, the role of MOFs in pancreatic disease management has undergone a remarkable transformation [37,38,39,40]. Initial investigations primarily focused on exploiting their high loading capacity for chemotherapeutic delivery. More recently, advances in materials engineering and mechanistic understanding have expanded MOF applications toward stromal remodeling, enhanced tumor penetration, ferroptosis- and cuproptosis-based precision therapy, energy-triggered therapeutic modalities, immune microenvironment reprogramming, and imaging-guided theranostics [41,42,43,44,45]. Importantly, the scope of MOF-based interventions has extended beyond pancreatic cancer to include inflammatory pancreatic disorders such as acute and chronic pancreatitis, highlighting their broader therapeutic potential [46,47]. These developments collectively reflect a paradigm shift in which MOFs are no longer viewed merely as delivery vehicles but rather as programmable platforms capable of orchestrating complex biological and therapeutic processes in a disease-specific manner [48,49,50].

Although numerous reviews have summarized the biomedical applications of MOFs in oncology, a comprehensive and disease-oriented perspective focusing specifically on pancreatic disorders remains lacking [51,52,53]. Moreover, the rapid emergence of catalytic nanotherapies, ferroptosis- and cuproptosis-oriented strategies, immune-regulatory MOFs, and multimodal theranostic systems has substantially reshaped the field in recent years [54]. Given the unique pathological characteristics of pancreatic diseases, including dense stromal barriers, hypoxia, immune suppression, and diagnostic difficulties, an updated and mechanistically integrated overview is urgently needed to elucidate how rational MOF engineering can address these challenges and accelerate clinical translation.

In this review, we systematically summarize recent advances in MOF-based technologies for pancreatic diseases from the perspectives of material design, biological mechanism, and translational potential (Figure 1). We first discuss the rational design principles underlying MOF construction and functional optimization. We then examine their applications in drug delivery and controlled release, tumor microenvironment remodeling, ferroptosis- and cuproptosis-oriented precision therapy, energy-triggered therapeutic platforms, cancer immunotherapy, and imaging-guided theranostics. Emerging applications in acute and chronic pancreatitis as well as MOF-assisted interventional therapies are subsequently highlighted. Finally, we critically evaluate the major challenges that currently hinder clinical translation.

Relevant literature was identified through searches of major scientific databases, including Web of Science, PubMed, and Google Scholar. The search strategy combined keywords related to metal–organic frameworks and pancreatic diseases, including “metal–organic frameworks”, “MOFs”, “pancreatic cancer”, “pancreatic ductal adenocarcinoma”, “pancreatitis”, “drug delivery”, “tumor microenvironment”, “immunotherapy”, “ferroptosis”, “cuproptosis”, “reactive oxygen species”, “imaging”, and “theranostics”. Publications focusing on MOF-based diagnosis, therapy, biological regulation, and translational development in pancreatic diseases were considered. Studies were selected based on their relevance to the central theme of this review, particularly those providing mechanistic insights, rational material design strategies, and representative biomedical applications. Additional relevant articles were identified through screening of references from selected publications to ensure comprehensive coverage of the rapidly evolving field.

## 2. Rational Design of MOFs for Pancreatic Diseases

The therapeutic performance of metalorganic frameworks (MOFs) in pancreatic diseases is fundamentally governed by their structural design and engineering strategies. Owing to their highly tunable composition, pore architecture, morphology, and physicochemical responsiveness, MOFs can be rationally tailored to address the major biological barriers encountered in pancreatic disease management, including insufficient imaging sensitivity, inefficient drug delivery, limited tumor penetration, immunosuppressive microenvironments, and poor spatiotemporal control of therapeutic payload release. Consequently, recent studies have increasingly focused on the development of multifunctional MOF systems through precise structural engineering, laying the foundation for subsequent applications in imaging, drug delivery, immunotherapy, and combination therapy (Table 1).

From a materials perspective, the biomedical performance of MOFs is closely associated with the coordinated contribution of their metal nodes, organic ligands, pore architecture, and physicochemical characteristics. Metal nodes and organic ligands determine the coordination environment, framework stability, catalytic or photophysical properties, and chemical responsiveness, whereas pore architecture governs surface area, molecular accommodation, cargo loading, and transport behavior. In parallel, particle size, morphology, surface chemistry, and stimulus responsiveness can further influence cellular uptake, tissue penetration, biodistribution, imaging performance, and controlled therapeutic release. Biodegradability should be considered as a structure- and environment-dependent property, because the degradation behavior of metal–ligand frameworks is affected by composition, particle size, morphology, and physiological conditions. Collectively, these interconnected features determine the biological fate and therapeutic performance of MOFs and provide the material basis for overcoming distinct biological barriers in pancreatic diseases.

Among various structural optimization approaches, dimensional engineering has emerged as an effective strategy for enhancing diagnostic performance. You et al. developed ultrathin Co–Mn-TCPP nanosheets through an ultrasound-assisted bottom-up assembly strategy, producing single-unit-cell MOF nanosheets with a thickness of approximately 1.82 nm (Figure 2a). Remarkably, the resulting two-dimensional architecture exhibited an ultrahigh longitudinal relaxivity (r_1_ = 12.05 mM^−1^ s^−1^), substantially outperforming many conventional MRI contrast agents. Mechanistic investigations revealed that the unique square-lattice topology effectively restricted molecular rotation and prolonged rotational correlation time, thereby amplifying proton relaxation efficiency. Such topology-driven engineering enabled highly sensitive magnetic resonance imaging and facilitated MRI-guided resection of small tumors, highlighting the importance of nanoscale structural control in the design of next-generation MOF-based imaging platforms [55].

Beyond morphology regulation, hierarchical integration of multiple MOF structures has become an attractive strategy for constructing multifunctional therapeutic systems. Liu et al. reported a controllable MOF-on-MOF growth approach in which doxorubicin-loaded ZIF-8 nanocrystals were assembled onto the surface of porphyrinic zirconium-based MOFs (Figure 2b) [56]. The thickness of the outer ZIF-8 shell could be precisely regulated, allowing simultaneous optimization of drug loading and stimulus-responsive release. Under acidic tumor microenvironment conditions, degradation of the ZIF-8 shell triggered doxorubicin release, whereas the porphyrin-containing MOF core generated singlet oxygen under light irradiation. This hierarchical architecture integrated chemotherapy and photodynamic therapy within a single nanoplatform, illustrating how rational hybrid engineering can endow MOFs with synergistic therapeutic capabilities that surpass those of individual components.

In parallel, optimization of drug encapsulation strategies has also become a critical aspect of MOF design. Traditional post-synthetic loading methods often suffer from limited reproducibility, low loading efficiency, and premature drug leakage. To overcome these limitations, Eskandari et al. introduced a one-pot synthetic strategy that enabled direct incorporation of hydrophobic natural compounds into UiO-66 during framework formation (Figure 2c) [57]. Compared with conventional post-synthetic loading approaches, this method achieved more homogeneous drug distribution throughout the MOF structure and significantly enhanced antitumor efficacy against three-dimensional pancreatic cancer spheroids. The study demonstrated that rational control of MOF synthesis routes can substantially influence drug-loading behavior and therapeutic outcomes, emphasizing the close relationship between framework construction and biological performance.

Besides improving drug delivery efficiency, contemporary MOF engineering increasingly incorporates tumor microenvironment-responsive functionalities. Dong et al. designed redox-responsive MOF nanocapsules capable of co-delivering doxorubicin and the IDO1 inhibitor NLG919 (Figure 2d) [58]. Following exposure to the reductive tumor microenvironment, the MOF framework underwent degradation and released both therapeutic agents. Doxorubicin induced immunogenic cell death, while NLG919 alleviated IDO1-mediated metabolic immunosuppression. Through simultaneous modulation of tumor metabolism and immune regulation, this platform effectively enhanced cytotoxic T-cell infiltration and reduced regulatory T-cell accumulation in pancreatic tumors. This work exemplifies how MOF design has evolved from passive drug carriers toward biologically interactive systems capable of reprogramming the tumor microenvironment.

Another major challenge in pancreatic cancer therapy is achieving precise and externally controllable drug release. To address this issue, Abdelhamid et al. constructed a magnetic-responsive MOF nanocomposite consisting of superparamagnetic iron oxide nanoparticles coated with a ZIF-8 shell and loaded with therapeutic cargo (Figure 2e) [59]. By applying either alternating magnetic fields or rotating magnetic fields, the platform could induce localized thermal effects or mechanical actuation, respectively, thereby triggering on-demand payload release. Importantly, both activation modes significantly reduced pancreatic cancer cell viability in two-dimensional cultures and three-dimensional heterospheroid models. This study highlights the growing trend toward integrating external physical stimuli into MOF design, enabling precise spatiotemporal regulation of therapeutic interventions.

Collectively, these representative studies illustrate the evolution of MOF engineering from simple framework synthesis toward sophisticated multifunctional platform design. Through topology regulation, hierarchical assembly, optimized drug encapsulation, microenvironment-responsive degradation, and externally triggered release mechanisms, MOFs have acquired increasingly diverse capabilities for pancreatic disease applications. These rational design principles not only underpin the therapeutic and diagnostic advances discussed in the following sections but also provide a conceptual framework for the development of next-generation precision nanomedicines.

## 3. MOFs for Drug Delivery and Controlled Release

Efficient drug delivery remains one of the most intensively investigated applications of metal–organic frameworks (MOFs) in pancreatic cancer therapy. The dense stromal architecture, elevated interstitial fluid pressure, and poor vascular perfusion characteristic of pancreatic tumors severely limit the accumulation and retention of conventional chemotherapeutic agents. Moreover, systemic administration of cytotoxic drugs frequently results in rapid drug clearance, burst release, and off-target toxicity. Owing to their high porosity, tunable pore environments, and versatile host–guest interactions, MOFs provide a unique platform for enhancing drug loading, protecting therapeutic cargos, and enabling controlled release. Recent advances have gradually shifted MOF-based drug delivery from simple encapsulation systems toward rationally engineered platforms capable of sustained release, synergistic therapy, and data-driven material optimization.

From a therapeutic perspective, MOF-based drug delivery strategies for pancreatic cancer can be broadly organized according to both the type of therapeutic cargo and the mechanism governing payload release. Chemotherapeutic agents, including gemcitabine, paclitaxel, doxorubicin, and SN-38, represent the most extensively investigated cargos and are primarily delivered to improve drug loading, retention, and controlled exposure within pancreatic tumors. Nucleic acid therapeutics, exemplified by siRNA co-delivery, provide an additional strategy for regulating tumor-associated molecular pathways and can be integrated with chemotherapy to achieve synergistic effects. Nucleic acid therapeutics, exemplified by siRNA co-delivery, provide an additional strategy for regulating tumor-associated molecular pathways and can be integrated with chemotherapy to achieve synergistic effects. MOF-based co-delivery of nucleic acid therapeutics with chemotherapeutic agents has also been explored, exemplified by a MOF-coated MnO_2_ nanosystem enabling co-delivery and intracellular activation of a surviving-targeting DNAzyme together with doxorubicin, thereby integrating gene silencing with chemotherapy [60]. Immunotherapeutic cargos and immune-modulating agents constitute another important category, although their applications are discussed in greater detail in Section 7. Beyond single-agent delivery, combination platforms integrate two or more therapeutic modalities, such as chemotherapy with photodynamic therapy or metabolic/immune modulation, to simultaneously target complementary mechanisms of tumor resistance. From the perspective of release regulation, MOF systems can further be classified as passive/sustained-release, tumor microenvironment-responsive, or externally triggered platforms, including pH-, redox-, and magnetic-responsive systems. Protein therapeutics and CRISPR-based cargo delivery represent emerging directions with considerable potential for MOF-mediated precision therapy; however, PDAC-specific MOF studies in these areas remain comparatively limited, highlighting an important opportunity for future investigation rather than a sufficiently mature category in the current literature.

One of the earliest challenges in pancreatic cancer chemotherapy is prolonging the release of frontline drugs such as gemcitabine. To address this issue, Panthi et al. developed mechanochemically synthesized binary and ternary complexes based on the aluminum MOF CYCU-3 and gemcitabine (Figure 3a) [61]. Through liquid-assisted grinding, gemcitabine was incorporated into the MOF framework via non-covalent interactions while largely preserving the crystal structure of CYCU-3. Compared with conventional formulations, the resulting complexes exhibited sustained gemcitabine release for up to 6000 min and produced a time-dependent suppression of PANC-1 pancreatic cancer cells. Notably, the incorporation of citronellol generated a ternary complex that maintained delayed drug-release characteristics, highlighting the flexibility of MOFs in accommodating multiple therapeutic components. This work demonstrates that rational control of MOF–drug interactions can effectively modulate release kinetics, thereby reducing burst release and potentially improving therapeutic safety.

Beyond functioning as passive drug reservoirs, MOFs can also actively participate in antitumor therapy through intrinsic biological activities. Luo et al. reported a series of transition metal-based imidazolyl benzoic acid MOFs (BMTA-1, BMTA-2, and BMTA-3), among which Co-bmta exhibited the strongest interaction with DNA and the most potent cytotoxicity against BxPC-3 pancreatic cancer cells (Figure 3b) [62]. Molecular docking studies suggested favorable DNA binding affinities, which were subsequently confirmed through experimental DNA interaction assays. Building upon these intrinsic therapeutic properties, gemcitabine was further loaded into the Co-bmta framework, resulting in the composite formulation Co-bmta@gem. The hybrid system achieved a high drug-loading efficiency of 81.86% and demonstrated enhanced apoptosis induction compared with either the MOF or gemcitabine alone. This study illustrates an important evolution in MOF-based drug delivery, whereby the framework itself contributes to anticancer activity while simultaneously serving as a carrier for chemotherapeutic agents, thereby enabling synergistic therapeutic effects.

While many studies have focused on empirical optimization of drug-loading systems, recent advances have introduced computational approaches to accelerate the rational design of MOF therapeutics. Melle et al. integrated machine learning with molecular simulations to screen a large MOF library for the encapsulation of clinically relevant pancreatic cancer drugs, including gemcitabine, paclitaxel, and SN-38 (Figure 3c) [63]. This data-driven workflow identified PCN-53 as a highly promising candidate based on its predicted drug–host interactions and loading capacity. Subsequent experimental validation confirmed efficient drug encapsulation, favorable biocompatibility, prolonged release behavior, and excellent colloidal stability over a 12-month period. Importantly, in vivo studies demonstrated that paclitaxel-loaded PCN-53 significantly suppressed primary tumor growth and metastatic dissemination, particularly when administered locally using a hydrogel delivery system. Unlike traditional trial-and-error material discovery strategies, this work established a predictive framework for MOF selection and optimization, representing a major step toward the clinical translation of MOF-based nanomedicines.

Taken together, these studies highlight the progressive evolution of MOF-based drug delivery systems in pancreatic cancer. Early efforts focused primarily on extending drug release through host–guest interactions, whereas subsequent developments incorporated intrinsic therapeutic functionality and synergistic drug-loading strategies. More recently, the emergence of machine learning-assisted material screening has transformed MOF design from empirical experimentation into a predictive and rational process. Collectively, these advances demonstrate that MOFs are not merely passive carriers but increasingly sophisticated therapeutic platforms capable of addressing multiple pharmacological limitations simultaneously. Such progress provides a critical foundation for the subsequent development of microenvironment-responsive and penetration-enhancing MOF systems discussed in the following section.

## 4. Tumor Microenvironment Remodeling and Tumor Penetration

Pancreatic ductal adenocarcinoma (PDAC) is characterized by one of the most complex and treatment-refractory tumor microenvironments among solid malignancies. A hallmark feature of PDAC is the extensive desmoplastic stroma, which consists of abundant extracellular matrix (ECM) components, activated fibroblasts, immunosuppressive cells, and aberrant vasculature. This stromal barrier can account for the majority of tumor volume and substantially impedes the intratumoral transport of therapeutic agents, thereby limiting the efficacy of both conventional chemotherapy and nanomedicine-based interventions. Consequently, increasing attention has been directed toward the development of MOF-based platforms capable of actively remodeling the tumor microenvironment rather than merely delivering therapeutic cargos. By degrading extracellular matrix components, reprogramming stromal cells, and enhancing immune cell infiltration, these systems offer a promising strategy for overcoming one of the principal barriers to effective pancreatic cancer treatment.

Desmoplasia in PDAC represents a dynamic stromal process involving pancreatic stellate cell activation, extracellular matrix (ECM) remodeling, excessive collagen deposition, and accumulation of hyaluronic acid (HA). These alterations increase stromal density and tissue resistance to molecular transport and can contribute to elevated interstitial pressure, thereby limiting the penetration of therapeutic agents and immune cells. Accordingly, effective stromal remodeling requires coordinated regulation of ECM components and stromal cellular activity. MOF-based platforms offer several potential approaches to address these barriers. As discussed below, collagenase-loaded MOFs can directly degrade collagen-rich ECM, while stromal-regulating MOF systems can reduce collagen deposition. In addition, HAase-releasing MOF systems can degrade HA and reduce stromal density, facilitating deeper therapeutic and immune-cell infiltration.

Direct modulation of the extracellular matrix represents an intuitive and highly effective approach for improving intratumoral transport. Liu et al. developed a tumor-targeted collagenase-encapsulating metal–organic framework (PMOCol) through a protein–metal ion–ligand coordination strategy, enabling exceptionally high enzyme loading efficiency while maintaining enzymatic activity (Figure 4a) [64]. Unlike conventional protein-loaded MOFs that often suffer from low encapsulation efficiency and potential metal-associated toxicity, PMOCol achieved a loading capacity of 80.3 wt% and exhibited improved biocompatibility. Following accumulation within the tumor microenvironment, collagenase was released in an active form and selectively degraded extracellular matrix components, thereby alleviating the dense stromal barrier that restricts drug diffusion and immune-cell infiltration. Importantly, ECM remodeling facilitated deeper penetration of therapeutic agents and enhanced infiltration of immune effector cells into pancreatic tumors. When combined with a near-infrared photothermal component, the platform further induced immunogenic tumor cell death and generated systemic antitumor immune memory, demonstrating that stromal remodeling can simultaneously improve both drug delivery and immunotherapeutic efficacy. This study highlights the potential of MOFs as multifunctional carriers capable of integrating matrix degradation with immune activation, thereby transforming the hostile pancreatic tumor microenvironment into a more therapeutically accessible state.

While enzymatic degradation directly targets extracellular matrix architecture, reprogramming stromal cellular components represents another complementary strategy for enhancing tumor penetration. Huang et al. developed an iron-containing MOF-coated nanoplatform (PTFE) designed to simultaneously regulate the stromal microenvironment and induce ferroptotic tumor cell death (Figure 4b) [65]. The system consisted of an erastin-loaded PLGA core surrounded by a metal–phenolic MOF shell formed through the coordination of Fe^3+^ and tannic acid. Notably, PTFE exhibited the ability to repolarize tumor-associated macrophages toward a pro-inflammatory M1 phenotype, thereby indirectly suppressing the activation of tumor-associated fibroblasts and reducing collagen deposition, thereby alleviating the collagen-rich stromal barrier within pancreatic tumors. This stromal normalization effect substantially improved nanoparticle accumulation and deep tumor penetration in both multicellular tumor spheroid models and highly desmoplastic pancreatic tumor models in vivo. In addition to its stromal remodeling capacity, the released iron ions promoted Fenton chemistry while erastin triggered ferroptosis, jointly disrupting intracellular redox homeostasis and overcoming therapeutic resistance. These findings underscore the importance of integrating microenvironment regulation with tumoricidal mechanisms, as improved penetration alone may be insufficient without effective downstream therapeutic responses.

Although PMOCol and PTFE employ distinct mechanisms, they collectively illustrate a broader paradigm shift in MOF-based pancreatic cancer therapy. Traditional nanomedicine strategies primarily focus on increasing drug accumulation within tumors; however, the exceptional stromal complexity of pancreatic cancer frequently prevents therapeutics from reaching malignant cells even after successful tumor localization. In contrast, both platforms recognize the tumor microenvironment itself as a therapeutic target. PMOCol achieves this through direct enzymatic degradation of extracellular matrix barriers, whereas PTFE accomplishes stromal remodeling by altering the phenotype of key cellular regulators within the tumor niche. Despite their mechanistic differences, both approaches converge on the same therapeutic objective: enhancing intratumoral penetration and improving access of therapeutic agents and immune cells to otherwise inaccessible tumor regions.

Collectively, these studies demonstrate that successful pancreatic cancer nanomedicine requires not only efficient drug delivery but also active remodeling of the tumor microenvironment. By targeting extracellular matrix components, stromal fibroblasts, and tumor-associated immune cells, MOF-based platforms can effectively dismantle the biological barriers that limit therapeutic efficacy. Such microenvironment-oriented strategies provide a critical foundation for the advanced therapeutic modalities discussed in subsequent sections, particularly those involving ferroptosis, cuproptosis, and energy-triggered therapeutic systems that require efficient intratumoral distribution to achieve maximal efficacy.

Beyond stromal desmoplasia, the pancreatic tumor microenvironment is characterized by a network of interconnected abnormalities, including hypoxia, metabolic reprogramming, acidic pH, redox imbalance, immune suppression, and vascular dysfunction. These features are not independent barriers but mutually reinforce one another to promote therapeutic resistance and limit intratumoral drug distribution. MOF engineering provides complementary strategies to address these pathological constraints. Hypoxia can be accommodated through oxygen-independent sonodynamic or radical-generating systems, as exemplified by MOF-derived TiO_2_/C and hypoxia-responsive Cu-MOF/AIPH platforms. Metabolic and redox vulnerabilities can be exploited by redox-responsive MOFs, ferroptosis-inducing systems, and platforms that disrupt glutathione or cysteine metabolism. Acidic pH can serve as an endogenous trigger for MOF degradation and cargo release, enabling selective activation within the tumor microenvironment. Meanwhile, immune suppression can be alleviated through coordinated regulation of tumor immunogenicity, immune-cell recruitment, stromal remodeling, and immune checkpoint pathways, as discussed in Section 7. Severe vascular abnormalities and hypovascularization further restrict perfusion and intratumoral transport; although current MOF studies have primarily addressed this limitation indirectly by remodeling stromal barriers and improving nanoparticle distribution and penetration, direct vascular normalization remains insufficiently established. Collectively, these examples highlight the potential of MOFs to integrate multiple microenvironment-responsive functions rather than targeting a single pathological feature in isolation.

## 5. Ferroptosis- and Cuproptosis-Based Precision Therapy

Mechanistically, ferroptosis and cuproptosis represent distinct but metabolically interconnected forms of regulated cell death that exploit vulnerabilities in iron, copper, lipid, antioxidant, and mitochondrial homeostasis. Ferroptosis is critically associated with disruption of intracellular iron homeostasis and iron-dependent oxidative reactions, which promote phospholipid peroxidation when cellular antioxidant defenses become insufficient. In PDAC, the cysteine–glutathione (GSH) axis constitutes an important protective network against ferroptotic stress, whereas suppression of GSH metabolism and glutathione peroxidase 4 (GPX4) activity can facilitate the accumulation of lipid peroxides and drive ferroptotic cell death. Accordingly, MOF-based systems can induce ferroptosis by increasing bioavailable iron and catalytic ROS generation, restricting cysteine/GSH metabolism, suppressing GPX4 activity, or combining these mechanisms with therapeutic stimuli. Cuproptosis, in contrast, is characterized by copper-dependent mitochondrial proteotoxic stress associated with the lipoylation of mitochondrial proteins and disruption of mitochondrial homeostasis. In PDAC, dysregulated copper availability and signaling pathways associated with cuproptosis resistance, including the SERPINB3–MAPK–FDX1 axis, may create exploitable metabolic vulnerabilities. MOF platforms can therefore provide a localized copper source while simultaneously modulating resistance pathways, thereby restoring sensitivity to cuproptosis. Importantly, these mechanisms are therapeutically relevant to PDAC because they target metabolic and stress-adaptation networks that contribute to resistance to conventional apoptosis-centered therapies, providing complementary routes for overcoming ferroptosis and cuproptosis resistance.

Pancreatic ductal adenocarcinoma (PDAC) exhibits profound metabolic plasticity and adaptive stress-response mechanisms, rendering conventional apoptosis-centered therapies insufficient for durable tumor control. In recent years, ferroptosis and cuproptosis have emerged as two highly promising forms of regulated cell death that exploit metabolic vulnerabilities distinct from apoptosis, necroptosis, and autophagy [66]. Owing to their tunable composition, redox activity, and cargo-loading capability, MOFs have become attractive platforms for simultaneously inducing oxidative stress, disrupting antioxidant defenses, and integrating imaging functions, thereby enabling precision regulation of ferroptotic and cuproptotic pathways in pancreatic cancer. As illustrated in Figure 5, current MOF-based strategies have evolved from simple ROS amplification toward sophisticated interventions targeting ferroptosis resistance networks, mitochondrial metabolic regulation, and copper-dependent cell death mechanisms.

Beyond direct tumor cell elimination, emerging evidence suggests that ferroptosis and cuproptosis may also influence the immune landscape of pancreatic tumors. Ferroptotic cell death is increasingly recognized as an immunologically relevant process because oxidative lipid damage and subsequent release of damage-associated molecular patterns (DAMPs) may enhance tumor antigen exposure and modulate immune-cell recruitment. Therefore, MOF platforms capable of inducing ferroptosis may not only overcome apoptosis resistance but also provide opportunities to improve immune recognition and synergize with immunotherapeutic strategies.

Similarly, cuproptosis represents a mitochondria-associated metabolic vulnerability that is closely linked with copper homeostasis and cellular metabolic regulation. By disrupting mitochondrial protein function and metabolic adaptation, cuproptosis-inducing MOF systems may reshape tumor metabolic states and potentially influence the interaction between tumor cells and immune components within the tumor microenvironment. These findings highlight that ferroptosis- and cuproptosis-oriented MOF therapies should not be considered isolated cytotoxic strategies, but rather multifunctional approaches capable of integrating metabolic intervention, tumor killing, and immune microenvironment remodeling.

A representative example is the CD133-targeted manganese–porphyrin MOF theranostic platform (MEC) developed by Xu et al. (Figure 5a) [67]. Unlike earlier ferroptosis-inducing systems that primarily focused on tumor-cell intrinsic antioxidant pathways, this study recognized that pancreatic cancer ferroptosis resistance is simultaneously sustained by both tumor cells and cancer-associated fibroblasts (CAFs). Specifically, CAFs establish an alternative cysteine supply pathway through the TGFβ1–Cys–GSH axis, enabling persistent glutathione (GSH) synthesis and thereby attenuating ferroptotic responses. Because GSH-dependent antioxidant defense is closely linked to the detoxification of lipid peroxides, persistent GSH availability contributes to ferroptosis resistance. To overcome this challenge, the authors proposed a dual-pathway blockade strategy in which a manganese-porphyrin MOF loaded with erastin and modified with anti-CD133 antibodies selectively targets PDAC stem-like cells while disrupting cysteine metabolism in both tumor cells and CAFs. Upon tumor accumulation, the platform simultaneously inhibits GSH synthesis, promotes Mn-mediated Fenton-like catalysis, and enhances sonodynamic ROS production through the porphyrinic framework. The resulting amplification of lipid peroxidation effectively overcomes ferroptosis resistance. Importantly, the manganese-containing MOF also provides magnetic resonance imaging capability, enabling real-time visualization of intratumoral redox status and therapeutic responses. This work highlights an important conceptual shift in ferroptosis therapy—from targeting cancer cells alone toward comprehensive regulation of the tumor–stroma metabolic network.

This system therefore simultaneously disrupts the antioxidant and iron-dependent arms of ferroptotic defense while introducing mitochondrial stress as an additional amplification mechanism. While the MEC platform focuses on overcoming ferroptosis resistance, another emerging direction involves exploiting mitochondrial dysfunction as a central driver of ferroptotic death. Xu et al. developed a multifunctional nanocomposite, CaO_2_@Fe(SS)-MOF@Ce6@PAA (CFMCP), that integrates calcium overload, photodynamic therapy (PDT), and chemodynamic therapy (CDT) into a single MOF-based system (Figure 5b) [68]. In this design, the Fe-containing MOF framework functions not only as a carrier for calcium peroxide nanoparticles and the photosensitizer chlorin e6, but also as a catalytic center for Fenton chemistry. Following cellular internalization, the platform depletes intracellular GSH, suppresses GPX4 activity, and elevates intracellular Fe^2+^ levels, thereby creating conditions favorable for ferroptosis induction. More importantly, sustained Ca^2+^ release triggers mitochondrial calcium overload, resulting in severe mitochondrial dysfunction and oxidative stress amplification. Mechanistic studies demonstrated that inhibition of mitochondrial calcium uptake significantly attenuated ferroptotic cell death, confirming the critical role of mitochondrial Ca^2+^ dysregulation in this process. Compared with conventional ferroptosis strategies that primarily rely on iron-dependent ROS generation, this approach introduces mitochondrial calcium metabolism as an additional therapeutic lever, thereby broadening the mechanistic landscape of MOF-mediated ferroptosis therapy.

Beyond ferroptosis, cuproptosis has recently emerged as a distinct copper-dependent cell death modality characterized by mitochondrial protein lipoylation and proteotoxic stress. Thus, cuproptosis sensitivity depends not only on copper availability but also on the cellular machinery governing copper-dependent mitochondrial proteotoxic stress. The discovery of cuproptosis has generated considerable interest because pancreatic cancer exhibits profound metabolic heterogeneity and frequently develops resistance to traditional cell death pathways. Huang et al. identified the SERPINB3–MAPK signaling axis as a critical regulator of cuproptosis resistance in pancreatic cancer and subsequently developed a MOF-based strategy to overcome this resistance (Figure 5c) [69]. Their study revealed that SERPINB3 suppresses the expression of ferredoxin 1 (FDX1), a key mediator of cuproptosis, through sustained activation of MAPK signaling. Based on this mechanistic insight, the authors engineered a multifunctional MOF capable of co-delivering copper ions and a MEK inhibitor. This platform simultaneously restores cuproptosis sensitivity through MAPK inhibition while providing the copper source required for cuproptotic activation. Notably, the therapeutic effect extends beyond direct tumor cell killing. The induction of cuproptosis also enhances intratumoral CD8^+^ T-cell infiltration and synergizes with immune checkpoint blockade, establishing a mechanistic link between metal-dependent cell death and antitumor immunity. This study exemplifies the transition of MOF-based therapeutics from passive delivery vehicles to mechanism-guided precision nanomedicines capable of targeting specific resistance pathways.

Collectively, these studies demonstrate the rapid evolution of MOF-enabled precision therapy in pancreatic cancer. Early ferroptosis-oriented platforms mainly focused on ROS amplification and iron metabolism regulation; however, recent advances increasingly emphasize overcoming resistance mechanisms embedded within the tumor microenvironment, including CAF-mediated metabolic support, mitochondrial adaptive responses, and oncogenic signaling networks. Meanwhile, the emergence of cuproptosis introduces an entirely new therapeutic paradigm that leverages copper homeostasis and mitochondrial metabolism as actionable vulnerabilities. Early ferroptosis-oriented platforms mainly focused on ROS amplification and iron metabolism regulation; however, recent advances increasingly emphasize overcoming resistance mechanisms embedded within the tumor microenvironment, including metabolic support from cancer-associated fibroblasts and complex crosstalk between tumor and stromal or immune cells [70]. Importantly, MOFs provide a unique structural framework capable of integrating metal ions, therapeutic cargos, imaging agents, and targeting ligands within a single nanosystem. Such multifunctionality enables simultaneous diagnosis, therapy, and mechanistic intervention, distinguishing MOF-based platforms from conventional nanomedicines. Looking forward, rationally engineered MOFs capable of co-regulating ferroptosis, cuproptosis, and antitumor immunity may offer particularly promising opportunities for overcoming the profound therapeutic resistance that characterizes pancreatic cancer.

## 6. Energy-Triggered Therapeutic Platforms

Energy-triggered therapeutic strategies have emerged as an important direction for overcoming the intrinsic limitations of conventional pancreatic cancer treatments. Unlike passive drug delivery systems, these approaches utilize externally applied or internally generated energy to activate therapeutic responses in a spatiotemporally controlled manner. Among them, sonodynamic therapy (SDT), chemodynamic therapy (CDT), and their synergistic combinations have attracted substantial attention owing to their ability to induce reactive oxygen species (ROS)-mediated tumor destruction while minimizing systemic toxicity. Importantly, the structural versatility of metal–organic frameworks (MOFs) enables the integration of catalytic centers, sonosensitizers, radical initiators, and imaging components within a single platform, making MOFs particularly suitable for energy-triggered precision therapy. As summarized in Figure 6, recent studies have progressively evolved from enhancing ROS generation efficiency to overcoming hypoxia-associated therapeutic resistance and constructing multifunctional oxygen-independent therapeutic systems.

Mechanistically, ROS-mediated therapies can be distinguished according to the energy input or catalytic process responsible for ROS generation. Photodynamic therapy (PDT) relies on light-activated photosensitizers to generate cytotoxic ROS, whereas chemodynamic therapy (CDT) exploits endogenous chemical reactions, typically metal-mediated Fenton or Fenton-like chemistry, to convert intracellular substrates such as H_2_O_2_ into highly reactive species. Sonodynamic therapy (SDT) replaces light irradiation with ultrasound activation and can generate type I and/or type II ROS, offering a deeper tissue penetration advantage over light-dependent approaches. Radiodynamic therapy (RDT) represents a distinct radiation-triggered strategy in which ionizing radiation activates ROS-generating or radiosensitizing components. Importantly, the ^125^I-MIL-88B (Fe) system discussed below is more appropriately classified as a radiocatalytic enhancement of CDT rather than canonical RDT, because the radionuclide-derived hydrated electrons primarily accelerate Fe^3+^/Fe^2+^ cycling and Fenton catalysis [71]. In parallel, catalytic nanozyme activity differs from externally triggered PDT, SDT, or RDT because enzyme-mimetic metal centers can catalyze ROS-generating reactions using endogenous substrates without requiring external energy input. These modalities therefore exhibit complementary therapeutic characteristics: PDT provides efficient localized ROS generation but may be constrained by light penetration and oxygen availability; CDT and nanozyme-like catalysis are intrinsically activated within the tumor but depend on substrate availability, catalytic metal cycling, and the physicochemical conditions of the tumor microenvironment; SDT offers superior tissue penetration but conventional type II pathways remain vulnerable to hypoxia; and RDT may provide deep-tissue activation but remains comparatively underexplored in MOF-based PDAC therapy. Rational MOF engineering can mitigate these limitations by integrating photosensitizers, catalytic metal centers, sonosensitizers, radical initiators, or radiation-responsive components within a single platform.

One representative strategy focuses on improving the efficiency of chemodynamic therapy by overcoming the rate-limiting valence transition of catalytic metal ions. Wang et al. developed a radioiodine-labeled iron-based MOF nanoparticle, ^125^I-MIL-88B (Fe), which exploits radionuclide-derived hydrated electrons to accelerate Fe^3+^/Fe^2+^ conversion and thereby amplify Fenton catalysis (Figure 6a) [71]. Traditional CDT efficacy is often constrained by the slow reduction of Fe^3+^ into catalytically active Fe^2+^, resulting in insufficient hydroxyl radical (•OH) production within tumors. In this system, the incorporated radioisotope ^125^I continuously generates hydrated electrons (eaq−) in aqueous environments, facilitating rapid Fe^3+^/Fe^2+^ cycling and sustained ROS generation. Simultaneously, the porous MIL-88B(Fe) framework enriches endogenous H_2_O_2_ and serves as an efficient catalytic reactor for hydroxyl radical production. Both in vitro and in vivo studies demonstrated significantly enhanced CDT efficacy through activation of the ROS/MAPK/p53 signaling axis. More importantly, this work introduces a novel concept in which radionuclides function not merely as imaging or radiotherapeutic agents but also as electron donors that directly regulate catalytic reactions. Such a strategy expands the functional repertoire of MOF-based CDT and establishes a new paradigm for integrating nuclear medicine with nanocatalytic therapy.

While CDT relies primarily on endogenous hydrogen peroxide as a substrate, sonodynamic therapy utilizes ultrasound energy to trigger ROS generation with superior tissue penetration compared with light-based modalities. However, the severe hypoxia characteristic of pancreatic tumors substantially limits the efficacy of conventional type II SDT, which depends on molecular oxygen for singlet oxygen production. To address this challenge, Cao et al. designed a MOF-derived tablet-like TiO_2_/C nanocomposite capable of mediating oxygen-independent type I SDT (Figure 6b) [72]. The carbon matrix derived from the MOF precursor enhances electron electron-transfer efficiency and stabilizes the TiO_2_ structure under repeated ultrasound irradiation. Consequently, the nanocomposite maintains robust ROS production even under hypoxic conditions and after multiple treatment cycles. Unlike traditional oxygen-dependent sonosensitizers, TiO_2_/C generates cytotoxic radicals through oxygen-independent pathways, thereby overcoming one of the major biological barriers associated with pancreatic cancer therapy. The ability to sustain repeated ROS generation without significant loss of activity highlights the importance of MOF-derived structural engineering in improving SDT durability and therapeutic reliability.

Importantly, the therapeutic potential of ultrasound-triggered ROS generation has also been demonstrated in non-MOF nanoplatforms. A hollow mesoporous carbon-based sono-immunotherapy system generated ROS under ultrasound irradiation and enhanced antitumor immunity when combined with PD-L1 blockade, partly through activation of dendritic cells and remodeling of the immunosuppressive tumor microenvironment. This finding provides complementary evidence that sonodynamic therapy can be coupled with immune modulation, while also highlighting the broader therapeutic context in which MOF-based sonodynamic platforms may operate [75].

Building upon the concept of oxygen-independent sonodynamic therapy, Zhang et al. further enhanced therapeutic precision through the development of an ultra-small nucleus-targeted Ti-TCPP MOF sonosensitizer (Figure 6c) [73]. This platform addresses another key limitation of ROS-based therapies: the extremely short diffusion distance and lifespan of reactive oxygen species. Instead of relying solely on increasing ROS yield, the authors engineered a MOF capable of delivering ROS generation directly to the vicinity of genomic DNA. Following ultrasound irradiation, the Ti-TCPP MOF simultaneously generates both type I and type II ROS through oxygen-independent and oxygen-dependent pathways, respectively. Owing to its ultra-small size and intrinsic nucleus-targeting capability, the generated ROS can directly induce DNA damage and S-phase cell cycle arrest, leading to efficient tumor cell apoptosis. Notably, this study demonstrates that precise subcellular localization may be equally important as ROS production efficiency in determining SDT outcomes. The work therefore highlights how MOF engineering can be utilized not only to regulate ROS generation but also to optimize intracellular therapeutic distribution.

Beyond improving individual SDT systems, recent efforts have increasingly focused on constructing multifunctional platforms that combine complementary ROS-generating mechanisms. A representative example is the hypoxia-responsive Cu-MOF/AIPH nanoplatform developed by Sun et al. for synergistic sono-chemodynamic therapy (Figure 6d) [74]. In this design, the copper-based MOF serves multiple functions simultaneously. Under hypoxic tumor conditions, the framework undergoes degradation, releasing Cu^2+^ ions and the free-radical initiator AIPH. The released Cu^2+^ depletes intracellular glutathione and is reduced to Cu^+^, which subsequently catalyzes Fenton-like reactions for chemodynamic therapy. Concurrently, ultrasound irradiation triggers AIPH decomposition, generating alkyl radicals in an oxygen-independent manner. These radicals directly induce DNA damage and apoptosis regardless of oxygen availability. Furthermore, nitrogen bubbles generated during AIPH decomposition enhance intratumoral penetration and improve nanoparticle distribution throughout the dense pancreatic tumor microenvironment. Compared with single-modality SDT or CDT approaches, this strategy demonstrates how MOFs can function as highly adaptable platforms for integrating multiple ROS-generating pathways into a coordinated therapeutic system capable of overcoming hypoxia-associated resistance.

Collectively, these studies illustrate the rapid evolution of MOF-based energy-triggered therapies from simple ROS-generating nanoplatforms toward highly sophisticated systems capable of regulating catalytic reactions, overcoming hypoxia, achieving subcellular targeting, and integrating multiple therapeutic modalities. A common theme emerging from these investigations is that therapeutic efficacy is increasingly determined not merely by the quantity of ROS generated but by the ability to precisely control when, where, and how reactive species are produced within the tumor microenvironment. MOFs are uniquely positioned to address these challenges because their tunable composition, porosity, and structural versatility allow simultaneous incorporation of catalytic metals, sonosensitizers, radionuclides, and responsive therapeutic cargos. Looking forward, the integration of SDT, CDT, radiocatalytic strategies, and potentially RDT or other energy-responsive modalities within multifunctional MOF architectures may provide a promising strategy for overcoming the profound hypoxia, stromal barriers, and therapeutic resistance that characterize pancreatic cancer.

## 7. MOF-Enabled Cancer Immunotherapy

Immunotherapy has revolutionized the treatment landscape of multiple malignancies; however, its clinical efficacy in pancreatic cancer remains disappointingly limited. This limitation largely arises from the intrinsically low immunogenicity of pancreatic tumors, extensive stromal barriers, poor immune-cell infiltration, and the establishment of a profoundly immunosuppressive tumor microenvironment (TME). Consequently, contemporary immunotherapeutic strategies are increasingly shifting from simply activating immune cells toward comprehensive reprogramming of tumor–immune interactions [76]. Owing to their programmable architectures, high cargo-loading capacity, and responsiveness to tumor-specific stimuli, MOFs have emerged as versatile platforms capable of simultaneously modulating antigen presentation, innate immune activation, stromal remodeling, and adoptive cell therapy. As illustrated in Figure 7, recent MOF-based immunotherapeutic systems have evolved from enhancing tumor immunogenicity to orchestrating systemic antitumor immune responses and overcoming multiple barriers that restrict immune-cell infiltration.

From an immunological perspective, MOF-based immunotherapy can be viewed as a coordinated intervention across multiple components of the pancreatic tumor immune microenvironment. These include tumor-associated macrophages (TAMs), T cells, dendritic cells (DCs), immune checkpoint pathways, myeloid-derived suppressor cells (MDSCs), and cancer-associated fibroblasts (CAFs). Among these components, macrophage polarization and T-cell function are directly addressed by current PDAC-specific MOF studies. For example, MOF-mediated stromal remodeling can promote TAM repolarization toward a pro-inflammatory M1 phenotype, while acid-responsive MOF systems combined with TIL therapy can enhance T-cell recruitment, infiltration, and persistence while limiting T-cell exhaustion. Immune checkpoint blockade, particularly PD-1/PD-L1-directed strategies, can further sustain cytotoxic T-cell activity and cooperate with MOF-mediated innate immune activation. In parallel, enhancement of antigen presentation represents an important interface between MOF-induced tumor immunogenicity and dendritic-cell-mediated adaptive immune priming. However, direct MOF-based regulation of DC maturation and MDSC abundance or function remains comparatively underexplored in PDAC, highlighting important opportunities for future immune-engineering strategies. CAFs represent another critical stromal component because their activation and extracellular matrix production contribute to immune exclusion and impaired therapeutic penetration; accordingly, MOF-mediated CAF suppression and extracellular matrix remodeling may simultaneously improve drug transport and immune-cell access. Collectively, these strategies indicate that the therapeutic potential of MOFs in pancreatic cancer lies not simply in activating individual immune effectors, but in remodeling the interconnected cellular and stromal networks that maintain an immunosuppressive tumor microenvironment.

One emerging strategy focuses on addressing the fundamental problem of low tumor immunogenicity. Pancreatic cancer is typically characterized by a relatively low tumor mutation burden (TMB) and limited neoantigen expression, resulting in insufficient immune recognition and poor responsiveness to immunotherapy. To overcome this challenge, Wang et al. developed a DNA stability-intervening nanoregulator, termed MOFDOX&siATR, which simultaneously induces DNA damage and suppresses DNA repair pathways to enhance neoantigen generation (Figure 7a) [77]. The platform consists of a MOF carrier co-delivering doxorubicin (DOX), a DNA-damaging agent, and siRNA targeting ATR, a key regulator of DNA damage repair. Through concurrent induction of genomic instability and inhibition of DNA repair, the system effectively increases mutation accumulation within tumor cells, thereby elevating the probability of neoantigen formation. Notably, the authors demonstrated that this strategy altered tumor gene-expression profiles and promoted the generation of the neoantigen Atp8b1, ultimately enhancing the immunogenicity of pancreatic tumors. This work is conceptually significant because it shifts the focus of nanomedicine-enabled immunotherapy from immune activation alone toward active engineering of tumor antigenicity. In essence, MOFs are utilized not merely as delivery vehicles but as tools for reshaping the evolutionary landscape of tumor cells to render them more visible to the immune system.

While increasing antigenicity improves the likelihood of immune recognition, effective antitumor immunity also requires robust activation of innate immune pathways capable of initiating systemic immune responses. In this regard, Luo et al. developed a STING agonist-conjugated MOF platform (GA-MOF) designed to synergize with radiotherapy and promote immune activation throughout the tumor microenvironment (Figure 7b) [78]. Radiotherapy has long been recognized as a means of inducing immunogenic cell death (ICD), yet its ability to generate durable systemic antitumor immunity remains limited by insufficient immune stimulation. To address this issue, the authors conjugated the cyclic dinucleotide STING agonist 2′,3′-cGAMP onto MOF nanoradiosensitizers, thereby integrating radiosensitization with innate immune activation. Following irradiation, GA-MOF not only enhanced local tumor destruction but also promoted the formation of immune-cell-rich structures termed artificial leukocytoid structures, which subsequently evolved into highly immunostimulatory immune hotspots. These structures facilitated antigen presentation, immune-cell recruitment, and sustained activation of antitumor immunity. Importantly, when combined with immune checkpoint blockade, the platform generated systemic immune responses capable of suppressing distant untreated tumors, demonstrating a pronounced abscopal effect. Compared with conventional nanoradiosensitizers that primarily focus on enhancing radiation-induced cytotoxicity, GA-MOF highlights the growing role of MOFs as immune-engineering platforms that actively coordinate interactions between radiotherapy and the immune system.

Despite advances in antigen generation and immune activation, the dense desmoplastic stroma characteristic of pancreatic cancer remains a major physical barrier that restricts immune-cell infiltration and limits the efficacy of adoptive cellular therapies. To address this challenge, Gan et al. developed an αPD-1-conjugated acid-responsive ZIF-8 nanodrug integrated with tumor-infiltrating lymphocyte (TIL) therapy (Figure 7c) [79]. This innovative platform tackles several key obstacles simultaneously, including stromal obstruction, insufficient T-cell trafficking, and T-cell exhaustion. The ZIF-8 nanoparticles were engineered to release hyaluronidase (HAase) and decitabine (DEC) selectively within the acidic tumor microenvironment. HAase-mediated degradation of hyaluronic acid (HA), a major component of the extracellular matrix, reduced stromal density, whereas DEC promoted the secretion of CCL5, a chemokine that recruits additional T cells into tumors. Simultaneously, αPD-1 antibodies remained conjugated to the TIL surface, preventing exhaustion and sustaining cytotoxic activity. Remarkably, this design established a positive feedback infiltration loop whereby newly recruited T cells delivered additional nanodrugs, further amplifying stromal remodeling and immune-cell recruitment. As a result, TIL infiltration increased dramatically, enabling efficient tumor eradication and suppression of metastasis at substantially reduced cell doses. This study exemplifies a new generation of MOF-assisted cellular immunotherapies in which nanomaterials actively reshape the tumor microenvironment to maximize the therapeutic potential of immune cells.

Taken together, these studies illustrate a clear evolution in the design philosophy of MOF-based immunotherapy for pancreatic cancer. Early efforts primarily focused on delivering immunomodulatory agents, whereas contemporary strategies increasingly address the multifaceted barriers that underpin immune resistance. Specifically, MOFs are now being employed to enhance tumor antigenicity through genomic regulation, activate innate immune pathways via STING signaling and radiotherapy, and facilitate immune-cell infiltration through stromal remodeling and adoptive cell engineering. Importantly, these approaches target different yet complementary stages of the cancer–immunity cycle, ranging from antigen generation and presentation to immune-cell recruitment and effector function. Such convergence suggests that future MOF-based immunotherapies will likely evolve toward integrated platforms capable of simultaneously enhancing tumor immunogenicity, reprogramming the tumor microenvironment, and sustaining adaptive immune responses. Given the highly immunosuppressive nature of pancreatic cancer, these multifunctional MOF systems may provide a promising framework for transforming immunologically “cold” tumors into therapeutically responsive lesions and thereby unlocking the full potential of cancer immunotherapy.

## 8. MOF-Based Diagnosis, Imaging, and Theranostics

One of the most distinctive advantages of MOFs in pancreatic disease management lies in their ability to integrate diagnosis and therapy within a single platform. Beyond serving as drug carriers, MOFs can be engineered as imaging contrast agents, biosensing interfaces, and theranostic nanoplatforms owing to their tunable composition, porous architectures, and multifunctional surface chemistry. These features enable simultaneous disease detection, image-guided intervention, and therapeutic monitoring, which is particularly valuable for pancreatic cancer, where early diagnosis remains difficult and treatment response is highly heterogeneous.

From the perspective of imaging modality, MOF-enabled theranostic systems can be broadly classified into MRI, CT, PET, photoacoustic imaging (PAI), fluorescence imaging, and multimodal imaging platforms, each offering distinct advantages for pancreatic disease management. MRI provides high soft-tissue contrast and can be integrated with MOFs containing paramagnetic or magnetic components, making it particularly suitable for anatomical localization and longitudinal monitoring. CT offers high spatial resolution and rapid whole-body imaging, while the incorporation of high-Z elements into MOF-based platforms may further enhance X-ray attenuation and enable CT-guided intervention. PET provides highly sensitive and quantitative molecular imaging and is particularly valuable for whole-body assessment of tumor burden and treatment response, although the development of PET-active MOF systems specifically for pancreatic diseases remains comparatively limited. PAI combines optical contrast with ultrasound-based detection and therefore provides deeper tissue penetration than conventional fluorescence imaging while retaining molecular and functional imaging capabilities; MOF platforms can further integrate photoactive components to enable PAI-guided therapy. Fluorescence imaging offers high sensitivity and real-time visualization of nanoparticle distribution and therapeutic localization, but its limited tissue penetration restricts its application mainly to superficial or intraoperative settings. Accordingly, multimodal imaging strategies that combine complementary modalities, such as MRI/fluorescence or MRI/PAI/CT, can compensate for the limitations of individual techniques and provide anatomical, functional, and molecular information within a single platform. Importantly, imaging-guided theranostics extend beyond tumor visualization by enabling real-time assessment of nanoparticle accumulation, drug release, therapeutic response, and treatment-associated changes, thereby providing a foundation for image-guided intervention and longitudinal therapeutic monitoring.

Among theranostic systems, MOF-based nanoplatforms have demonstrated the capacity to combine targeted drug delivery with multimodal imaging guidance. Zhang et al. developed an iron-containing MOF nanocarrier (P@F-FD) co-loaded with doxorubicin and fluorescent probes and further coated with polydopamine to achieve pH-responsive drug release and dual-mode imaging (Figure 8a) [80]. Following accumulation within tumor tissues, the acidic pancreatic tumor microenvironment triggered structural changes in the polydopamine shell, leading to controlled doxorubicin release. Simultaneously, the intrinsic iron components enabled magnetic resonance imaging (MRI), while the fluorescent module provided real-time optical tracking. This study exemplifies how MOF platforms can bridge therapeutic delivery and imaging navigation, facilitating more precise intervention and treatment monitoring. Importantly, such integrated systems address a key limitation of conventional chemotherapy, namely the inability to visualize drug distribution and therapeutic response in real time.

Beyond image-guided therapy, MOFs have also emerged as highly attractive materials for biomarker detection owing to their large surface area, abundant active sites, and excellent signal amplification capability. Carbohydrate antigen 19-9 (CA19-9), the most widely used clinical biomarker for pancreatic cancer, remains limited by insufficient sensitivity and specificity, particularly during early-stage disease. To improve CA19-9 detection performance, Chen et al. constructed an impedimetric immunosensor based on gold nanoparticle-supported HKUST-1 MOFs (Au@HKUST-1/SPCE) (Figure 8b) [81]. The hierarchical porous MOF structure provided abundant immobilization sites for biomolecular recognition and enhanced electron-transfer efficiency, enabling ultrasensitive detection of CA19-9 in serum with a detection limit below clinically relevant concentrations. This work highlights how MOF-assisted electrochemical sensing can substantially improve the analytical performance of traditional biomarker assays.

While CA19-9 remains the current clinical standard, emerging biomarkers may offer improved specificity for early pancreatic cancer detection. Small extracellular vesicles (sEVs), particularly glypican-1-positive sEVs (GPC-1^+^ sEVs), have attracted significant attention as potential early diagnostic biomarkers. To exploit this opportunity, Xi et al. developed an integrated real-time isolation and detection platform utilizing CD63 antibody-functionalized MOFs for efficient sEV capture and surface-enhanced Raman scattering (SERS)-based detection (Figure 8c) [82]. The MOF-based enrichment strategy achieved capture efficiencies approaching 94%, while the subsequent SERS analysis enabled highly sensitive quantification of GPC-1^+^ sEVs directly from patient serum samples. Notably, the platform completed isolation and detection within 35 min and successfully differentiated pancreatic cancer patients from healthy controls and patients with benign pancreatic lesions. Compared with conventional serum biomarkers, this approach illustrates the growing role of MOF-enabled liquid biopsy technologies for early disease diagnosis.

In parallel, advances in MOF engineering have enabled the development of highly conductive electrochemical interfaces for biomarker sensing. Palanisamy et al. reported a heterometallic Ni–Fe MOF grown directly on conductive nickel foam to construct a binder-free electrochemical immunosensor for CA19-9 detection (Figure 8d) [83]. The hierarchical porous structure and exposed bimetallic active sites significantly enhanced charge-transfer efficiency and antigen recognition performance. As a result, the sensor exhibited excellent selectivity, stability, and clinically relevant sensitivity in human serum samples. This work demonstrates how rational modulation of MOF composition can directly improve electrochemical sensing performance while simplifying device fabrication.

Further improvements in analytical sensitivity have been achieved through MOF-derived catalytic nanostructures. Fan et al. constructed an electrochemiluminescence (ECL) immunosensor employing MOF-templated FeCoS_2_ nano-octahedrons as coreactant enhancers and Zn^2+^-regulated CdS_γ_Se_1-γ_ nanospheres as ECL emitters (Figure 8e) [84]. The MOF-derived FeCoS_2_ architecture provided highly efficient catalytic activity through synergistic Fe–Co interactions, resulting in an ultrasensitive CA19-9 detection platform with an exceptionally low detection limit. Compared with conventional electrochemical assays, this strategy demonstrates the value of MOF-derived functional materials in signal amplification and ultrasensitive biomarker quantification.

Taken together, these studies reveal an important evolution in the role of MOFs within pancreatic cancer management. Early investigations primarily focused on utilizing MOFs as imaging contrast agents or drug carriers, whereas recent efforts increasingly emphasize integrated diagnostic–therapeutic systems and highly sensitive biomarker detection platforms. As illustrated in Figure 8, current MOF-based diagnostic technologies span multiple levels of disease assessment, including anatomical imaging, molecular imaging, serum biomarker quantification, extracellular vesicle profiling, and image-guided therapy. A comprehensive comparison of representative MOF-based theranostic platforms, including their structural characteristics, imaging modalities, therapeutic functions, biological mechanisms, and translational potential, is summarized in Table 2. This convergence of diagnostics and therapeutics reflects a broader trend toward precision oncology, where MOFs function not merely as passive carriers but as multifunctional platforms capable of disease detection, therapeutic delivery, treatment monitoring, and response prediction.

Nevertheless, despite remarkable advances in preclinical models, several challenges continue to hinder clinical translation. Imaging-oriented MOFs require further optimization regarding long-term biosafety, biodegradation kinetics, and imaging standardization. Similarly, MOF-based biosensors must demonstrate reproducibility, scalability, and robustness across large patient cohorts before clinical implementation. Future efforts will likely focus on integrating artificial intelligence-assisted biomarker analysis, multimodal imaging, and personalized therapeutic decision-making into unified MOF-enabled precision medicine platforms. Such developments may ultimately position MOFs as a key technological bridge connecting early diagnosis, real-time monitoring, and individualized treatment of pancreatic diseases.

## 9. Emerging Applications Beyond Conventional Cancer Therapy

### 9.1. MOFs for Acute and Chronic Pancreatitis

Although most current studies focus on pancreatic cancer, recent advances suggest that MOFs may also provide unique therapeutic opportunities for non-malignant pancreatic disorders, particularly acute pancreatitis (AP) and chronic pancreatitis (CP). Unlike pancreatic cancer, where therapeutic barriers primarily arise from stromal desmoplasia and immune suppression, pancreatitis is characterized by excessive oxidative stress, inflammatory cascades, acinar cell injury, and progressive fibrotic remodeling. Given their tunable catalytic activity and enzyme-mimicking properties, MOF-based nanozymes have emerged as attractive candidates for regulating these pathological processes.

Although PDAC remains the primary focus of current MOF-based pancreatic disease research, emerging studies indicate that MOFs may also provide valuable opportunities for addressing non-malignant pancreatic disorders, including acute pancreatitis, chronic pancreatitis, pancreatic fibrosis, and future regenerative applications. Unlike PDAC, where therapeutic barriers are dominated by stromal desmoplasia, immune suppression, and treatment resistance, inflammatory and fibrotic pancreatic diseases are primarily characterized by excessive oxidative stress, inflammatory amplification, abnormal extracellular matrix remodeling, and progressive tissue dysfunction. Owing to their tunable catalytic activity, antioxidant properties, and capacity for controlled therapeutic delivery, MOF-based systems may offer potential strategies for regulating these pathological processes. In acute and chronic pancreatitis, MOF-based nanozymes may help alleviate oxidative injury and inflammatory responses through enzyme-mimicking activities and microenvironment modulation. In pancreatic fibrosis, the ability of MOFs to regulate extracellular matrix-associated processes and stromal cell behavior suggests potential value for attenuating fibrotic remodeling. Furthermore, the combination of controlled bioactive factor delivery, oxidative stress regulation, and extracellular matrix modulation indicates that MOFs may represent promising platforms for future pancreatic tissue repair and regenerative medicine. However, compared with PDAC-focused applications, MOF-based strategies for non-malignant pancreatic diseases remain at an earlier developmental stage, and further investigation is required to clarify their biological mechanisms and translational potential.

Acute pancreatitis is strongly associated with excessive reactive oxygen species (ROS) accumulation, mitochondrial dysfunction, and inflammatory amplification. Therefore, developing catalytic nanomaterials capable of efficiently eliminating ROS represents a promising therapeutic strategy. Zhang et al. engineered a series of metal–-organic framework nanozymes with precisely controlled metal active centers and identified a Cu-based MOF exhibiting superior antioxidant activity compared with its Co- and Ni-based counterparts (Figure 9a) [85]. Owing to the biomimetic CuN_2_Cl_2_ catalytic site, the Cu-MOF displayed substantially enhanced superoxide dismutase (SOD)-like activity, catalase-like activity, and hydroxyl radical scavenging capability. Mechanistically, theoretical calculations demonstrated that the Cu coordination environment optimized substrate adsorption and facilitated catalytic turnover, thereby improving ROS elimination efficiency. Beyond direct antioxidant effects, this nanozyme activated the PINK1/PARK2-mediated mitophagy pathway, reducing both apoptosis and necrosis of pancreatic acinar cells. These findings highlight an emerging paradigm in which MOFs function not merely as drug carriers but as catalytic therapeutic agents capable of directly modulating disease-driving biochemical processes.

While acute pancreatitis is dominated by acute inflammatory injury, chronic pancreatitis is characterized by persistent inflammation, oxidative stress, and progressive pancreatic fibrosis. Given the central role of ROS in driving fibrogenesis, antioxidant nanotherapies have attracted increasing attention. Lai et al. developed a hyaluronic acid-coated cerium-based MOF nanozyme (HC@CeMOF) capable of selectively targeting inflamed pancreatic tissue through CD44-mediated macrophage recognition (Figure 9d) [88]. Following accumulation at inflammatory lesions, the CeMOF core scavenged ROS through reversible Ce^3+^/Ce^4+^ redox cycling, while encapsulated curcumin simultaneously suppressed NF-κB signaling and inflammatory cytokine production. The synergistic antioxidant and anti-inflammatory effects effectively inhibited pancreatic stellate cell activation and attenuated fibrosis progression. Compared with conventional anti-inflammatory therapies, this strategy illustrates how rationally engineered MOFs can integrate targeted delivery, catalytic ROS elimination, and immune modulation within a single therapeutic platform.

Collectively, these studies suggest that oxidative stress regulation represents a common mechanistic foundation underlying MOF-based interventions for both AP and CP. Importantly, unlike cancer-oriented applications that often rely on cytotoxicity induction, pancreatitis therapies emphasize restoring tissue homeostasis through antioxidant, anti-inflammatory, and regenerative mechanisms. This distinction substantially broadens the therapeutic landscape of MOFs within pancreatic diseases.

### 9.2. MOF-Assisted Interventional Therapy

Interventional therapies have become increasingly important for the management of locally advanced pancreatic cancer, particularly in patients who are not candidates for surgical resection. Among these approaches, irreversible electroporation (IRE) has gained considerable attention because it can ablate tumors while preserving critical vascular and ductal structures. However, conventional IRE remains limited by heterogeneous electric-field distribution, incomplete tumor ablation, and local recurrence. Recent studies indicate that MOFs can address these limitations by acting either as biological sensitizers or as functional materials that directly improve electric-field performance.

One strategy focuses on sensitizing tumor cells to electroporation-induced damage through ferroptosis induction. Li et al. demonstrated that iron-based MOF nanoparticles could promote intracellular iron accumulation and trigger ferroptotic susceptibility in pancreatic cancer cells (Figure 9b) [86]. However, compensatory upregulation of ferritin heavy chain 1 (FTH1) partially restored iron homeostasis and reduced therapeutic efficacy. To overcome this resistance mechanism, the authors developed a ROS-responsive nanomicelle encapsulating the FTH1-targeting PROTAC molecule C20U4V. When combined with MOF-Fe nanoparticles, this system effectively disrupted iron homeostasis, amplified lipid peroxidation, and significantly enhanced IRE-mediated tumor ablation. This work illustrates an important conceptual shift in which MOFs serve not only as drug delivery vehicles but also as biological amplifiers of interventional therapies through metabolic reprogramming and ferroptosis sensitization.

A complementary strategy involves directly integrating MOFs into the electroporation hardware itself. Xu et al. developed a conductive MOF-assisted bipolar IRE system that combines the cathode and anode into a single electrode architecture, thereby simplifying procedural implementation and improving electric-field uniformity (Figure 9c) [87]. The incorporation of conductive MOFs enhanced local electric-field distribution and increased tumor cell susceptibility to electrical ablation. Mechanistic studies demonstrated enhanced apoptosis induction and improved ablation consistency compared with conventional IRE systems. Both in vitro and in vivo evaluations confirmed substantial tumor suppression without significant toxicity. This approach represents a distinct application paradigm in which MOFs function as functional engineering materials that optimize device performance rather than acting as therapeutic cargo carriers.

Taken together, these studies reveal an emerging convergence between MOF nanotechnology and interventional oncology. Whereas traditional MOF research primarily emphasizes drug delivery and molecular therapy, recent developments suggest that MOFs can also enhance physical treatment modalities by modulating ferroptotic sensitivity, optimizing electric-field distribution, and improving ablation efficacy. Such integration may provide new opportunities for overcoming the limitations of current local therapies in pancreatic cancer and further expands the translational potential of MOFs beyond conventional nanomedicine applications.

## 10. Clinical Translation Challenges and Future Perspectives

Over the past decade, metal–organic frameworks (MOFs) have rapidly evolved from a class of porous coordination materials into highly versatile biomedical platforms for pancreatic disease management. As discussed throughout this review, rationally engineered MOFs have demonstrated remarkable capabilities in drug delivery, tumor microenvironment remodeling, ferroptosis and cuproptosis induction, energy-triggered therapy, cancer immunotherapy, molecular imaging, and integrated theranostics. More recently, their applications have expanded beyond conventional pancreatic cancer treatment to include acute and chronic pancreatitis as well as interventional therapeutic modalities. Collectively, these advances highlight a fundamental transition in the role of MOFs: from passive nanocarriers toward multifunctional disease-modulating systems capable of simultaneously sensing, responding to, and reshaping pathological microenvironments.

A central theme emerging from current research is that MOFs are uniquely positioned to address several hallmark challenges of pancreatic diseases. In pancreatic cancer, dense stromal barriers, profound hypoxia, metabolic reprogramming, immune suppression, and therapeutic resistance collectively contribute to poor clinical outcomes. Owing to their highly tunable compositions, hierarchical porosity, and programmable physicochemical properties, MOFs can be rationally designed to overcome multiple barriers simultaneously. This capability distinguishes MOFs from many conventional nanomedicines that typically address only a single therapeutic limitation. Particularly noteworthy is the growing convergence of multiple therapeutic mechanisms within individual MOF platforms, enabling the integration of targeted delivery, microenvironment modulation, cell death induction, imaging guidance, and immune activation. Such multifunctionality represents a promising direction for future precision medicine strategies in pancreatic diseases.

Despite these encouraging advances, substantial barriers remain before MOF-based technologies can achieve clinical translation. The first challenge concerns biosafety and biodegradability. Although many MOFs demonstrate favorable short-term biocompatibility in animal studies, their long-term biological fate remains insufficiently understood. The degradation kinetics of metal nodes and organic ligands may vary considerably depending on MOF composition, particle size, morphology, and physiological conditions. Potential accumulation of metal ions in the liver, spleen, kidney, or other organs raises concerns regarding chronic toxicity, particularly for MOFs containing transition metals with redox activity. Therefore, systematic investigations of pharmacokinetics, biodistribution, metabolism, clearance pathways, and long-term toxicological profiles are essential for future clinical development. These considerations further highlight that biodegradability and biological fate should be incorporated into MOF design at the materials-engineering stage rather than evaluated only during subsequent translational development.

Beyond general concerns regarding biosafety and biodegradation, the translational challenges of MOF-based pancreatic disease therapies are highly dependent on their specific functional designs. For drug-delivery-oriented MOFs, although high loading capacity and stimuli-responsive release provide substantial advantages, challenges remain in achieving reproducible large-scale synthesis, precise control of particle size and surface properties, and consistent batch-to-batch therapeutic performance. For MOF-based nanozyme and catalytic platforms, the therapeutic efficacy is often strongly influenced by the tumor microenvironment, including pH, oxygen availability, and endogenous substrate levels, which may result in variable responses among different tumor models and patients. Furthermore, imaging-guided theranostic MOFs face additional challenges related to long-term accumulation, imaging signal standardization, and correlation between imaging readouts and therapeutic outcomes.

Importantly, most current MOF-based platforms remain at the preclinical stage, and their clinical translation requires comprehensive evaluation of pharmacokinetics, biodistribution, immunological compatibility, manufacturing scalability, and regulatory compliance. Future development should therefore prioritize not only improving therapeutic efficacy but also establishing clinically relevant design criteria that integrate material performance, biological safety, and translational feasibility.

Beyond biological safety considerations, regulatory challenges represent another critical barrier for translating MOF-based nanomedicines from laboratory research into clinical applications. Unlike conventional small-molecule drugs, MOF platforms possess complex and tunable architectures, making their manufacturing consistency, quality control, and regulatory evaluation more challenging. From a chemistry, manufacturing, and controls (CMC) perspective, key considerations include reproducible synthesis, precise control of particle size and morphology, stability during storage, batch-to-batch consistency, and reliable characterization of structural and functional properties.

Furthermore, standardized characterization approaches are essential for establishing clear relationships between MOF structure and biological performance. Parameters including metal–ligand composition, crystallinity, porosity, surface modification, degradation behavior, drug-loading efficiency, and release profiles should be systematically evaluated to ensure product quality and reproducibility. In addition, the unique characteristics of MOF-based nanomedicines require appropriate adaptation of existing nanomedicine regulatory frameworks, particularly regarding long-term safety evaluation, biological fate, and clinical manufacturing requirements.

Therefore, future clinical translation of MOF-based pancreatic disease therapeutics will require interdisciplinary coordination among materials science, pharmaceutical development, regulatory science, and clinical medicine. Establishing standardized manufacturing protocols and comprehensive evaluation criteria will be essential for accelerating the transition of MOF platforms from promising experimental systems toward clinically approved nanomedicines.

Beyond biodegradation and metal ion toxicity, comprehensive biosafety evaluation of MOF-based biomedical platforms requires consideration of their entire biological fate after administration. The degradation process of MOFs involves not only the dissociation of metal nodes and organic ligands but also subsequent interactions between degradation products and biological systems. Parameters including particle size, surface modification, structural stability, and physiological microenvironment can collectively influence biodistribution, cellular uptake, metabolism, and clearance behavior. Therefore, systematic assessment of pharmacokinetics, organ accumulation, immune responses, and long-term tissue compatibility is essential for determining the translational feasibility of MOF-based nanomedicines.

In particular, the potential toxicity associated with released metal ions represents a critical consideration for MOF design. Different metal components may exhibit distinct biological behaviors, and excessive accumulation or prolonged exposure may induce unwanted effects in normal organs. In addition, interactions between MOF nanoparticles and immune components should be carefully evaluated, including potential immunogenicity and inflammatory responses. For pancreatic disease applications, special attention should also be given to interactions with normal pancreatic tissues, because therapeutic platforms should ideally achieve selective activity within pathological environments while minimizing disruption to healthy pancreatic cells and physiological functions.

Accordingly, future MOF development should incorporate biosafety considerations at the initial material-design stage rather than evaluating safety only after therapeutic performance has been established. Rational selection of metal nodes, organic ligands, particle size, degradation profiles, and surface functionalities will be critical for balancing therapeutic efficacy with biological compatibility and accelerating clinical translation.

A second challenge lies in manufacturing scalability and reproducibility. Most reported MOF systems are synthesized under highly controlled laboratory conditions using protocols that may be difficult to standardize for large-scale production. Variations in particle size distribution, crystallinity, drug loading efficiency, surface modification density, and batch-to-batch consistency can significantly influence biological performance. Beyond production-related challenges, regulatory evaluation and clinical readiness represent additional critical barriers for MOF-based pancreatic nanomedicine translation.

Beyond manufacturing challenges, regulatory evaluation and clinical readiness represent additional critical barriers for MOF-based pancreatic nanomedicine translation. Unlike conventional small-molecule drugs, MOF-based platforms possess complex compositions involving metal nodes, organic ligands, surface modifications, and therapeutic cargos, which complicates the establishment of standardized characterization criteria and regulatory assessment frameworks. Comprehensive evaluation of physicochemical properties, including particle size distribution, structural stability, degradation behavior, drug loading consistency, and impurity profiles, will be essential for defining quality-control standards. Furthermore, pharmacokinetic characteristics, including circulation stability, tissue distribution, metabolism, and clearance pathways, must be systematically investigated to determine clinical feasibility. From a translational perspective, future MOF development should prioritize clinically relevant manufacturing processes, reproducible production protocols, and integrated safety-efficacy evaluation strategies to facilitate progression from preclinical validation toward clinical application.

Another critical issue concerns biological complexity. The majority of current studies rely on cell lines or subcutaneous xenograft models, which incompletely recapitulate the unique biological characteristics of human pancreatic diseases. Pancreatic ductal adenocarcinoma exhibits extraordinary heterogeneity at the genetic, metabolic, stromal, and immunological levels. Similarly, chronic pancreatitis and acute pancreatitis involve highly dynamic inflammatory processes that are difficult to model experimentally. Future studies should therefore prioritize clinically relevant systems, including patient-derived organoids, genetically engineered mouse models, orthotopic tumor models, and humanized immune models, to improve translational predictability and therapeutic evaluation.

Future development of MOF-based pancreatic nanomedicine will likely be driven by the integration of intelligent material design, precision medicine, and advanced biological evaluation platforms. Artificial intelligence and machine learning-assisted approaches may accelerate MOF discovery by predicting structure–property relationships, optimizing metal–ligand combinations, and identifying material characteristics associated with improved therapeutic efficacy and biosafety. Such data-driven strategies may facilitate the transition from empirical material screening toward rational and predictive MOF engineering.

In parallel, future MOF platforms are expected to move toward patient-specific precision nanomedicine. By integrating disease-specific biomarkers, molecular profiling, and responsive therapeutic functions, MOFs may enable more individualized diagnosis and treatment strategies tailored to the heterogeneous characteristics of pancreatic diseases. Biomimetic MOF systems represent another promising direction, as the incorporation of biological components or cell-inspired functionalities may improve targeting capability, biocompatibility, and interaction with complex physiological environments.

Furthermore, advanced biological models, such as patient-derived organoids, may provide valuable platforms for evaluating MOF-based therapeutic responses in a more clinically relevant context. Organoid-guided screening could facilitate the assessment of therapeutic efficacy, drug sensitivity, and individualized treatment strategies before clinical application. Combined with multimodal imaging and therapeutic integration, next-generation MOF platforms may ultimately enable comprehensive pancreatic theranostic systems that achieve precise disease visualization, targeted intervention, and real-time therapeutic monitoring.

However, these emerging strategies remain at an early stage and require further validation regarding scalability, reproducibility, biosafety, and clinical feasibility. Future progress will depend on the convergence of materials science, artificial intelligence, biomedical engineering, and clinical research to transform MOF-based systems from experimental platforms into clinically applicable precision nanomedicines.

Another emerging opportunity lies in the convergence of diagnosis and therapy. Throughout this review, we have highlighted a growing trend toward MOF-based theranostic systems that combine molecular imaging, biomarker detection, drug delivery, and therapeutic monitoring within a single platform. Future developments may further integrate multimodal imaging, liquid biopsy technologies, and treatment-response prediction, enabling closed-loop precision medicine strategies for pancreatic diseases. Such systems could facilitate patient stratification, guide therapeutic decision-making, monitor treatment efficacy, and enable early detection of recurrence.

Beyond pancreatic cancer, the successful application of MOFs in acute pancreatitis, chronic pancreatitis, and interventional therapies suggests that their therapeutic scope may continue to expand. Given the shared pathological features among many pancreatic disorders—including oxidative stress, inflammation, fibrosis, immune dysregulation, and metabolic abnormalities—MOFs provide a highly adaptable platform for addressing diverse disease mechanisms. This broader perspective may ultimately establish MOFs as a unified technological framework for pancreatic disease management rather than as tools restricted to oncology alone.

In conclusion, MOF-based technologies have opened new avenues for the diagnosis and treatment of pancreatic diseases by enabling unprecedented control over drug delivery, microenvironment regulation, multimodal therapy, and precision imaging. While significant challenges remain regarding safety, manufacturing, and clinical translation, the rapid convergence of materials science, nanotechnology, oncology, immunology, and bioengineering is accelerating progress toward real-world applications. With continued interdisciplinary collaboration and increasing emphasis on translational research, MOFs are poised to evolve from promising experimental materials into clinically relevant platforms that may fundamentally reshape the future management of pancreatic diseases.

## 11. Conclusions

Metal–organic frameworks have emerged as a versatile and highly tunable platform for addressing the complex therapeutic challenges associated with pancreatic diseases. As summarized in this review, rationally engineered MOFs have demonstrated significant potential in pancreatic cancer management and other pancreatic disorders through their unique advantages in structural programmability, multifunctional integration, and responsive behavior. Their applications have expanded from conventional drug delivery systems toward advanced platforms capable of tumor microenvironment modulation, regulated cell death induction, immune regulation, molecular imaging, and integrated theranostic applications.

In particular, the ability of MOFs to precisely manipulate metal nodes, organic ligands, pore structures, surface functionalities, and therapeutic cargos enables the construction of customized nanoplatforms that address key pathological barriers of pancreatic diseases, including stromal obstruction, hypoxia, metabolic dysregulation, immune suppression, and therapeutic resistance. The convergence of MOF engineering with ferroptosis, cuproptosis, immunotherapy, and multimodal imaging further highlights their potential as multifunctional precision medicine platforms.

Despite these advances, several critical challenges remain before MOF-based technologies can achieve clinical translation, including comprehensive biosafety evaluation, predictable biodegradation, scalable manufacturing, standardized characterization, and validation in clinically relevant disease models. Future progress will require closer integration of materials science, biomedical engineering, and clinical research to establish reliable design principles that balance therapeutic efficacy with biological safety.

Overall, rationally engineered MOFs represent a promising technological framework for the next generation of pancreatic disease diagnosis and treatment. Continued advances in intelligent material design, precision medicine, and translational evaluation may further accelerate the development of MOF-based systems from experimental nanoplatforms toward clinically applicable therapeutic strategies.

## Figures and Tables

**Figure 1:**
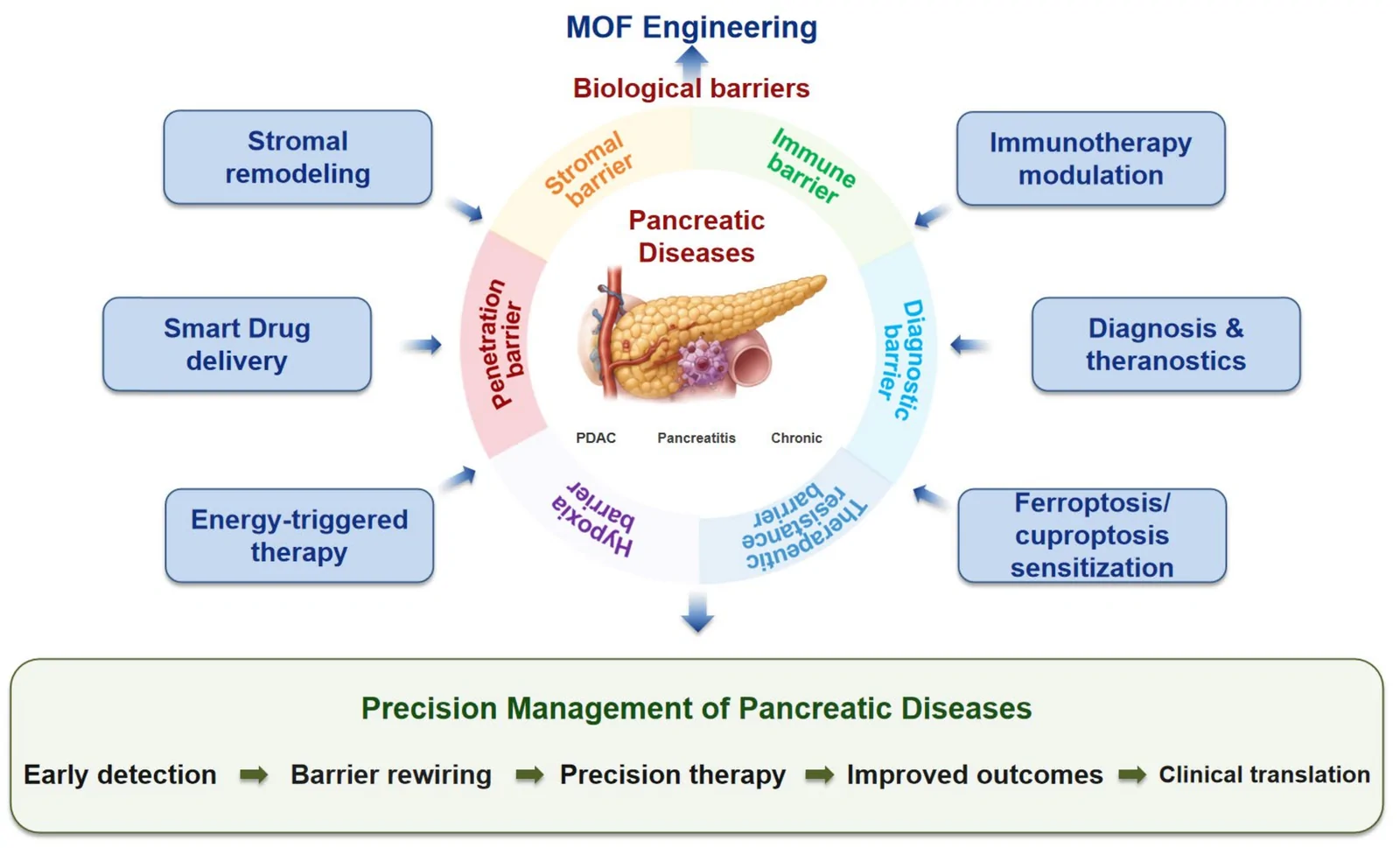
Conceptual framework of rationally engineered MOFs for overcoming biological barriers in pancreatic diseases.

**Figure 2:**
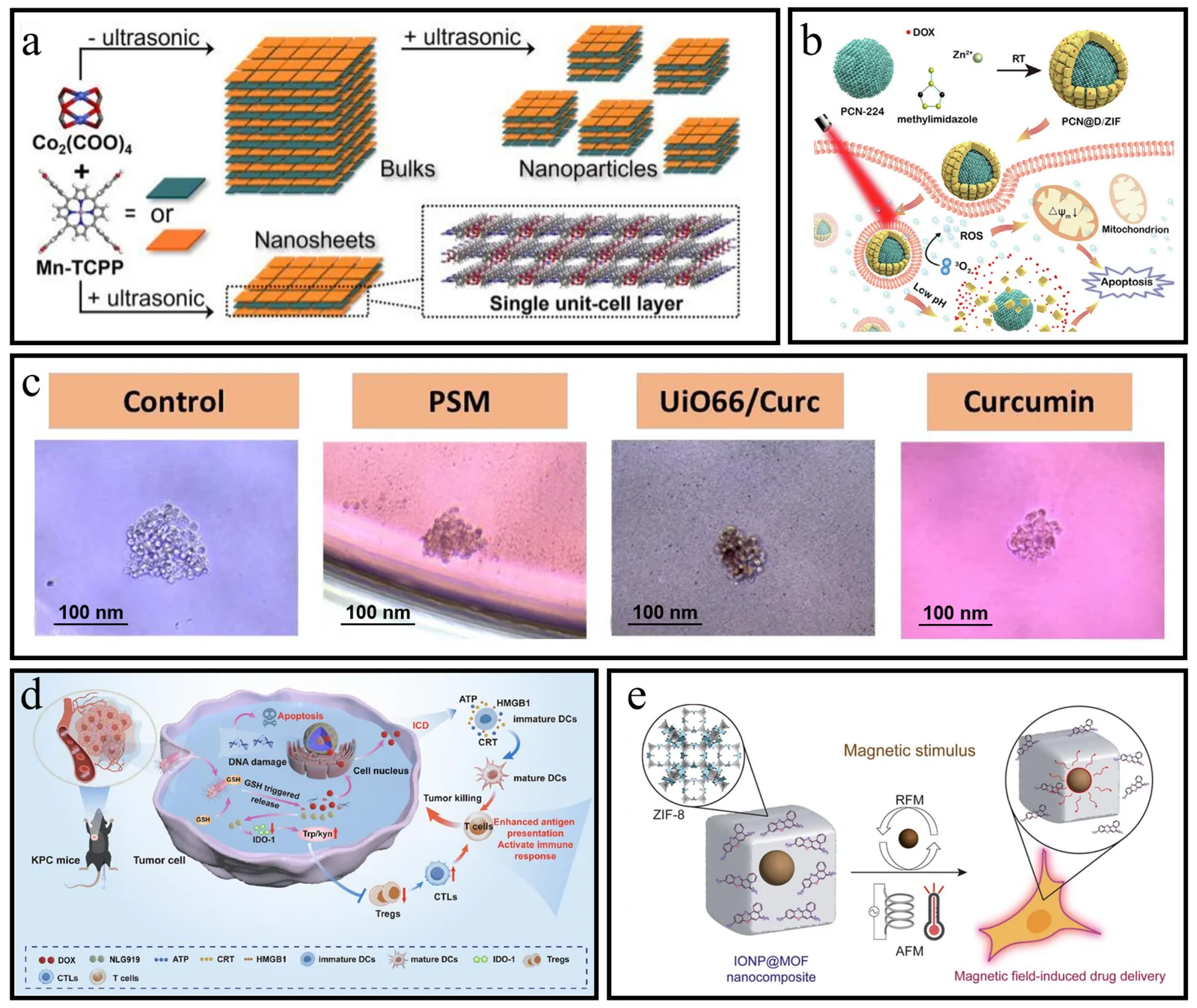
Rational design and smart engineering strategies of MOFs for pancreatic disease applications. (**a**) Schematic illustration of the ultrasound-assisted bottom-up synthesis of ultrathin CoMn-TCPP nanosheets with a single-unit-cell thickness [55]. (**b**) One-pot encapsulation of hydrophobic anticancer agents into UiO-66 through a solvothermal strategy for enhanced drug incorporation and distribution [56]. (**c**) Construction of a hybrid MOF-on-MOF architecture by the in situ growth of DOX-loaded ZIF-8 on porphyrinic Zr-MOFs for pH-responsive chemo-photodynamic therapy [57]. (**d**) Tumor microenvironment-responsive degradation of DNMCs enabling the co-delivery of doxorubicin and NLG919 for metabolic and immune modulation [58]. (**e**) Magnetic field-responsive ZIF-8 nanocomposites integrating superparamagnetic iron oxide nanoparticles for remotely controlled drug release under alternating or rotating magnetic fields [59].

**Figure 3:**
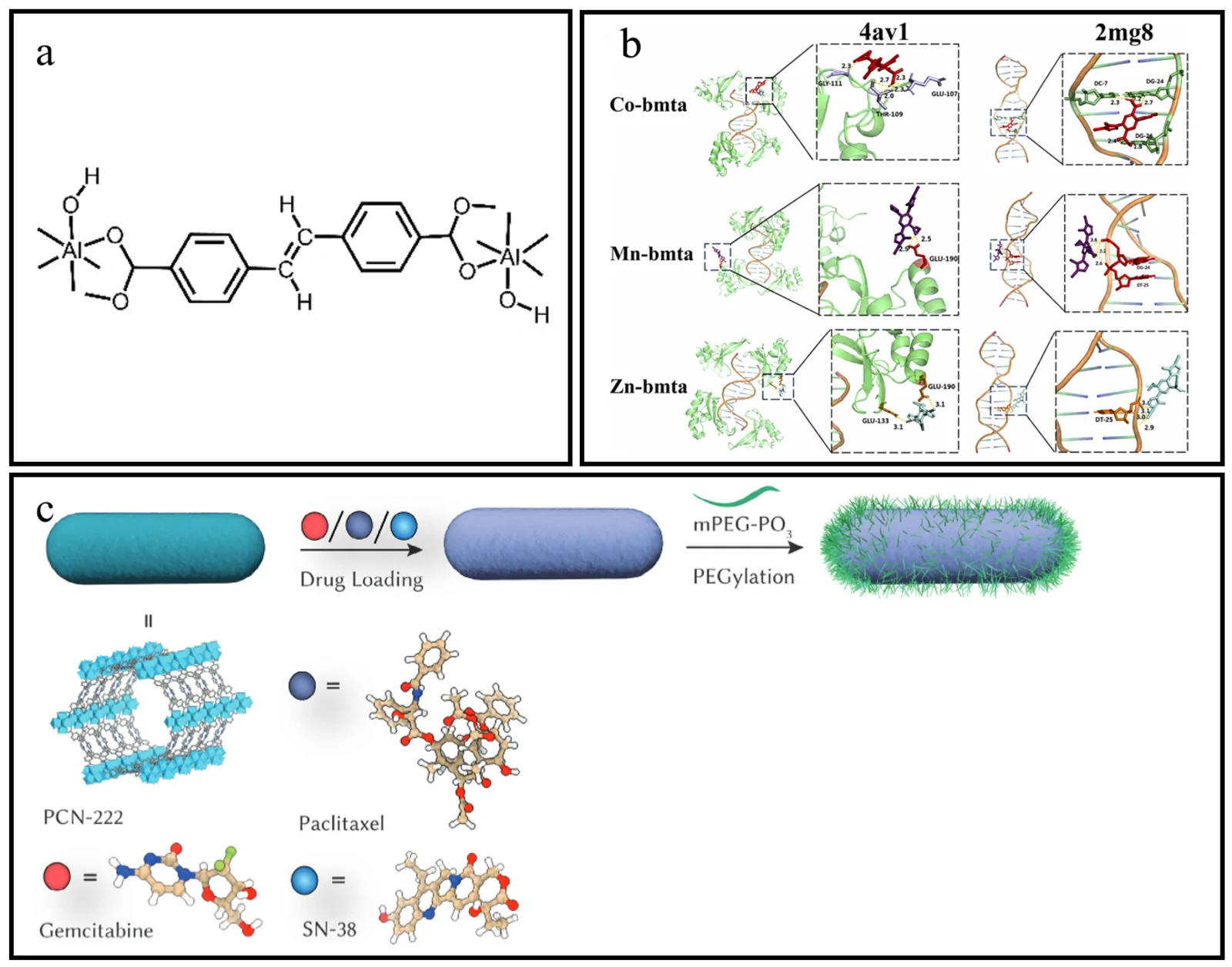
Representative MOF platforms for drug loading and delivery in pancreatic cancer. (**a**) Simplified two-dimensional secondary building unit (SBU) structure of the aluminum-based MOF CYCU-3 used for gemcitabine encapsulation and sustained drug release [61]. (**b**) Molecular docking models illustrating the interactions between DNA and the transition metal-based MOFs Co-bmta, Zn-bmta, and Mn-bmta [62]. (**c**) Chemical structures of PCN-53, gemcitabine, paclitaxel, and SN-38, together with a schematic illustration of drug loading and PEGylation of PCN-53 [63].

**Figure 4:**
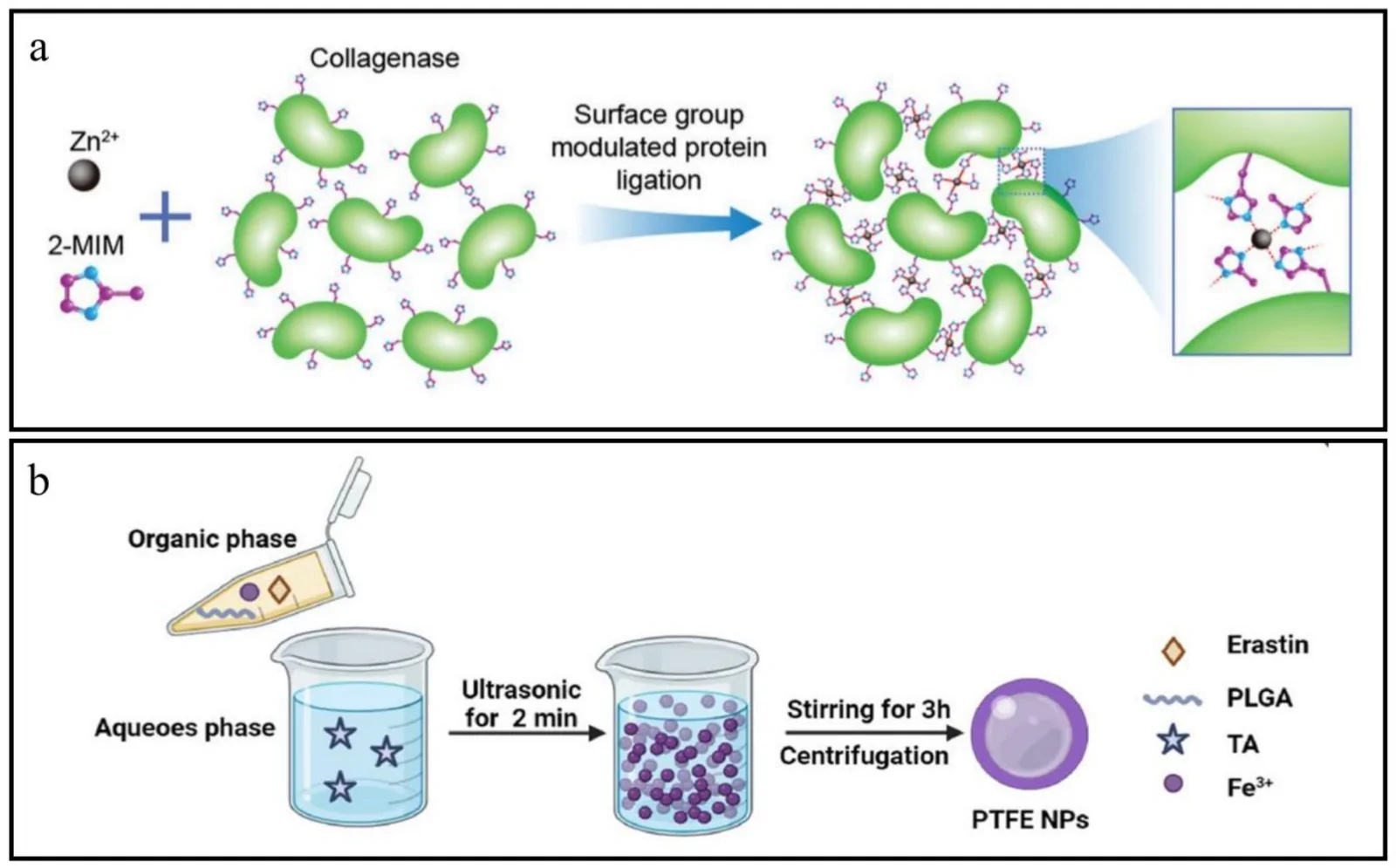
MOF-based strategies for stromal remodeling and enhanced intratumoral penetration in pancreatic cancer. (**a**) Schematic illustration of the synthesis process of collagenase-encapsulating PMOCol through protein–metal ion–ligand coordination for extracellular matrix regulation and photoimmunotherapy [64]. (**b**) Self-assembly fabrication of PTFE nanoparticles consisting of an erastin-loaded PLGA core and an Fe^3+^/tannic acid-coordinated MOF shell for stromal remodeling and ferroptosis-based therapy [65].

**Figure 5:**
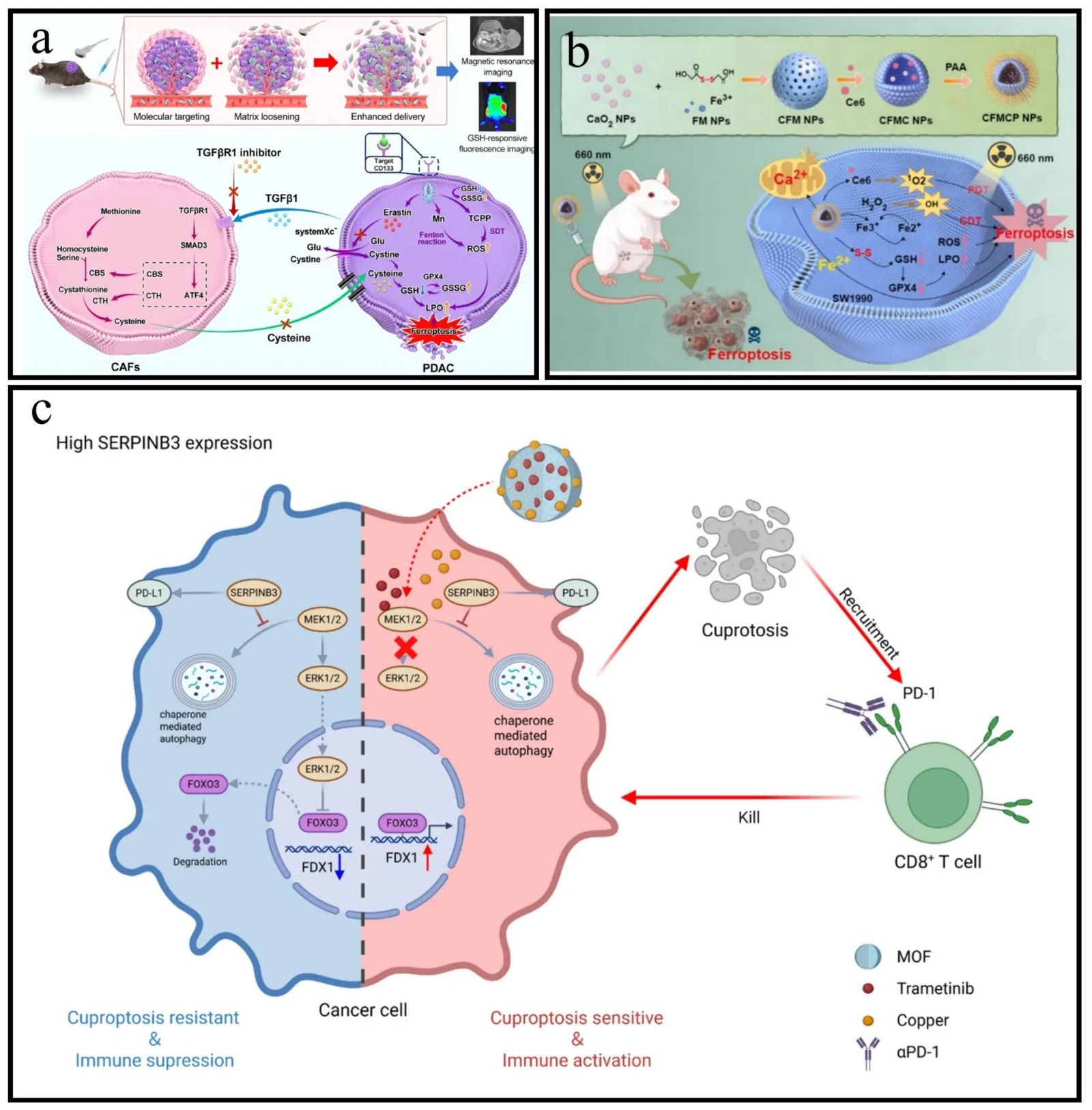
MOF-based ferroptosis- and cuproptosis-oriented therapeutic strategies for pancreatic cancer. (**a**) Schematic illustration of dual-pathway blockade targeting glutathione metabolism combined with sonodynamic/Fenton catalytic reactions to induce ferroptosis in pancreatic cancer [67]. (**b**) Multifunctional CFMCP nanoplatform integrating calcium overload, photodynamic therapy, and ferroptosis for synergistic pancreatic cancer treatment [68]. (**c**) MOF-mediated co-delivery of copper ions and a MEK inhibitor for cuproptosis induction and enhanced antitumor immunotherapy in SERPINB3-high pancreatic cancer [69].

**Figure 6:**
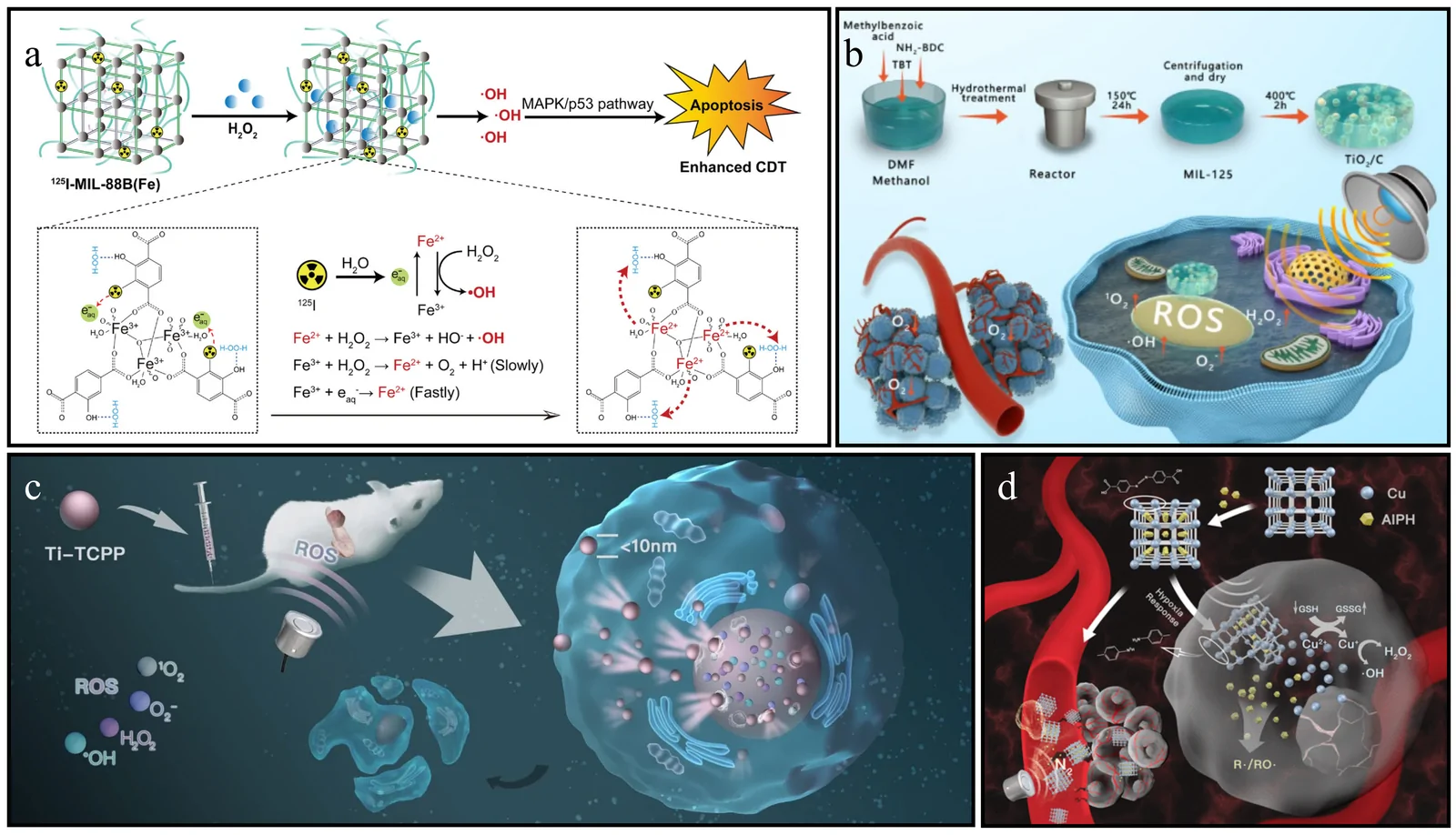
Representative MOF-based platforms for reactive oxygen species-mediated pancreatic cancer therapy. (**a**) Radioiodine-mediated enhancement of chemodynamic therapy using ^125^I-MIL-88B (Fe) nanoparticles through accelerated Fe^3+^/Fe^2+^ conversion and hydroxyl radical generation [71]. (**b**) MOF-derived TiO_2_/C nanocomposite for hypoxia-tolerant type I sonodynamic therapy under repeated ultrasound irradiation [72]. (**c**) Schematic illustration of nucleus-targeted ultra-small Ti-TCPP MOFs for sonodynamic therapy of pancreatic cancer [73]. (**d**) Structure of AIPH@Cu-MOF nanoparticles and the corresponding mechanism of hypoxia-responsive sono-chemodynamic therapy [74].

**Figure 7:**
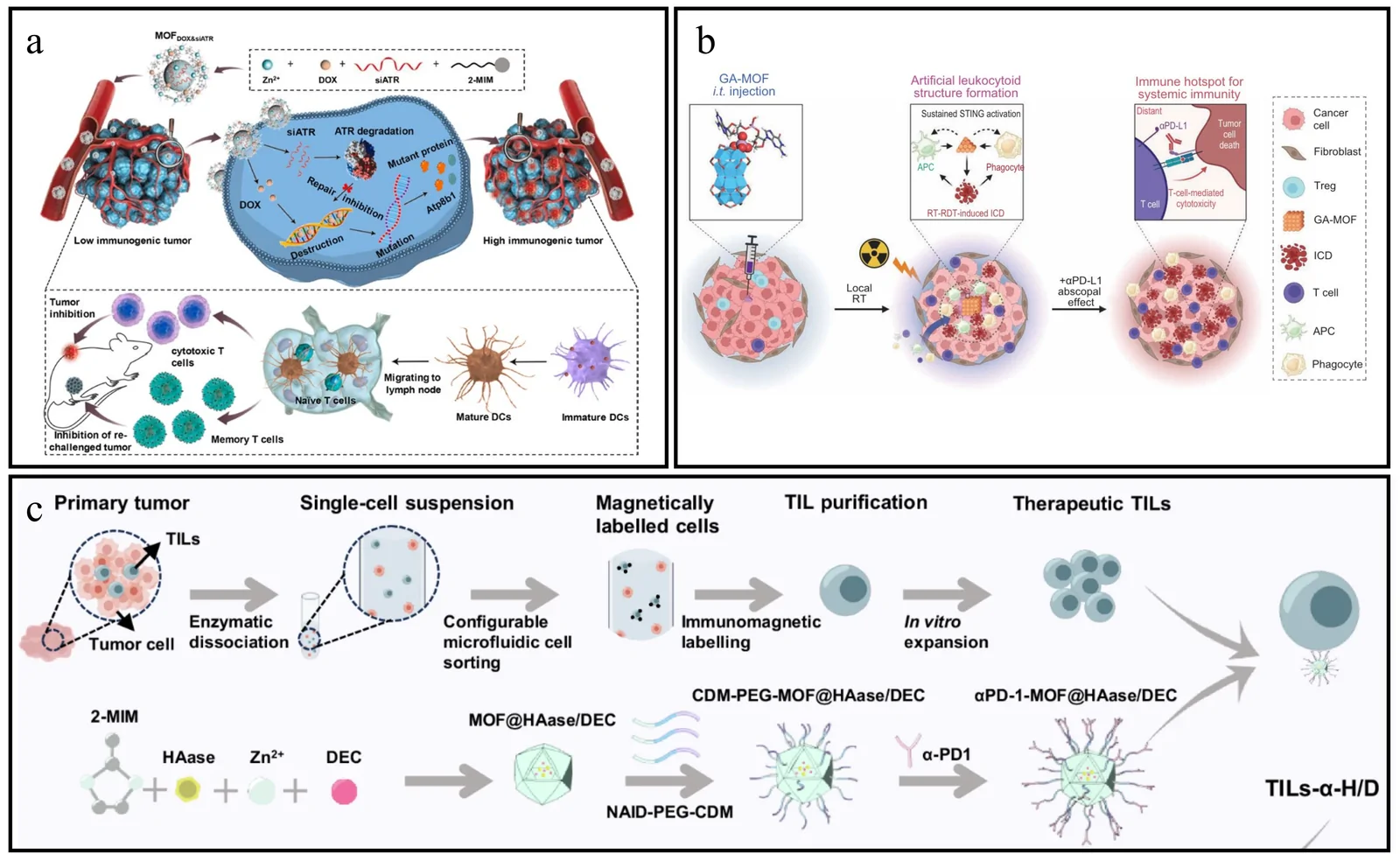
Representative MOF-based approaches for enhancing antitumor immunity in pancreatic cancer. (**a**) MOFDOX&siATR-mediated intervention of tumor DNA stability to promote neoantigen generation and immunogenic remodeling [77]. (**b**) GA-MOF-mediated activation of the STING pathway combined with radiotherapy-induced immunogenic cell death [78]. (**c**) Construction of αPD-1-conjugated nanodrug-loaded tumor-infiltrating lymphocytes for adoptive cellular immunotherapy [79].

**Figure 8:**
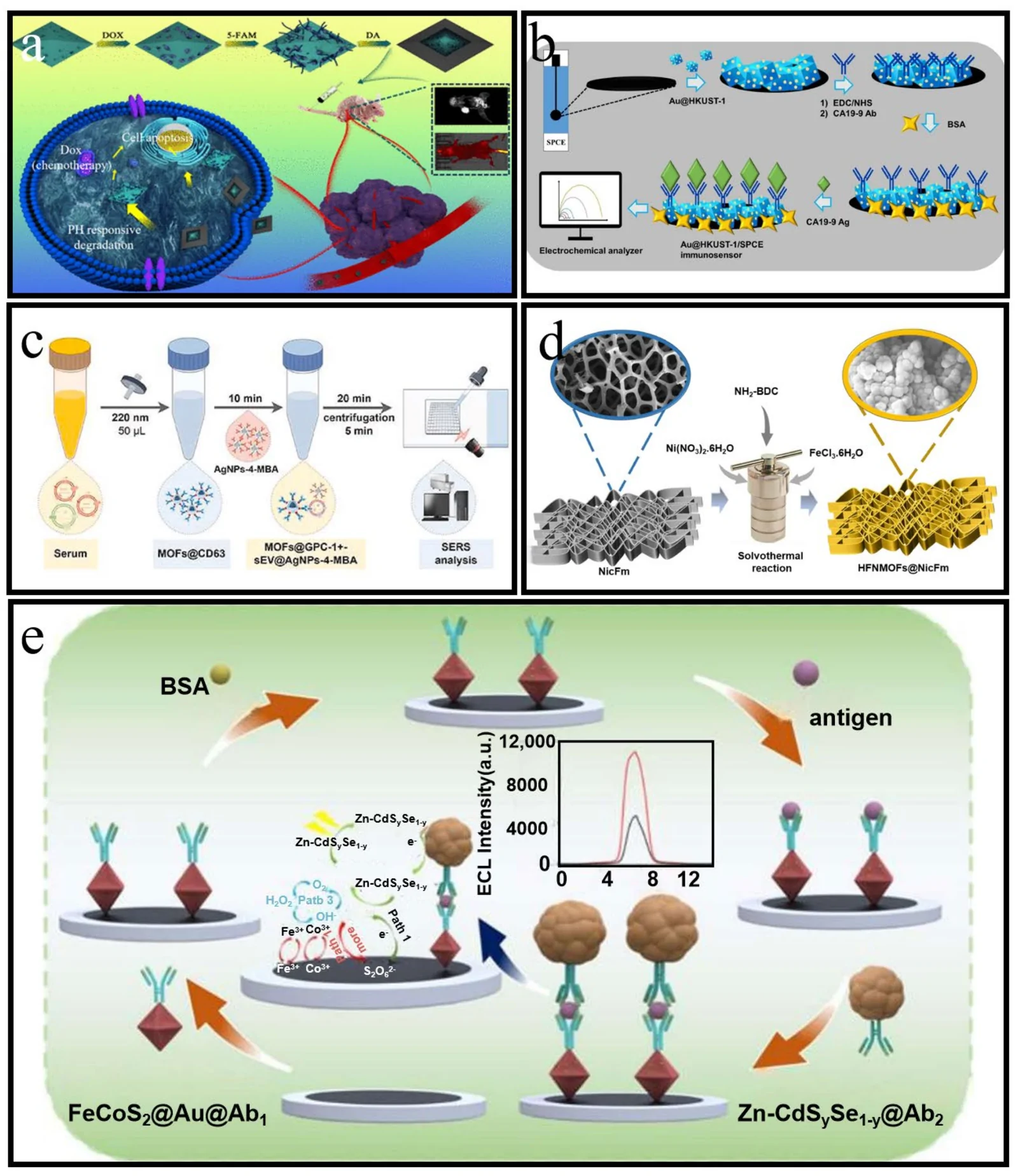
Representative MOF-based platforms for pancreatic cancer diagnosis and theranostics. (**a**) Schematic illustration of the synthesis and theranostic design of the MOF-based nanoparticle system P@F-FD for fluorescence-guided and MRI-assisted pancreatic cancer management [80]. (**b**) One-pot stepwise hydrothermal synthesis of the Au@HKUST-1 platform for electrochemical detection of the pancreatic cancer biomarker CA19-9 [81]. (**c**) Integrated real-time isolation and detection platform (IRTIDP) based on MOF-assisted enrichment and SERS analysis of glypican-1-positive small extracellular vesicles (GPC-1^+^-sEVs) [82]. (**d**) Solvothermal fabrication of heterometallic MOFs grown in situ on conductive nickel foam (HFNMOFs@NicFm) for electrochemical CA19-9 biosensing [83]. (**e**) Electrochemiluminescence immunosensor employing Zn^2+^-regulated CdS_γ_Se_1-γ_ nanospheres and MOF-derived FeCoS_2_ nano-octahedrons for ultrasensitive CA19-9 detection [84].

**Figure 9:**
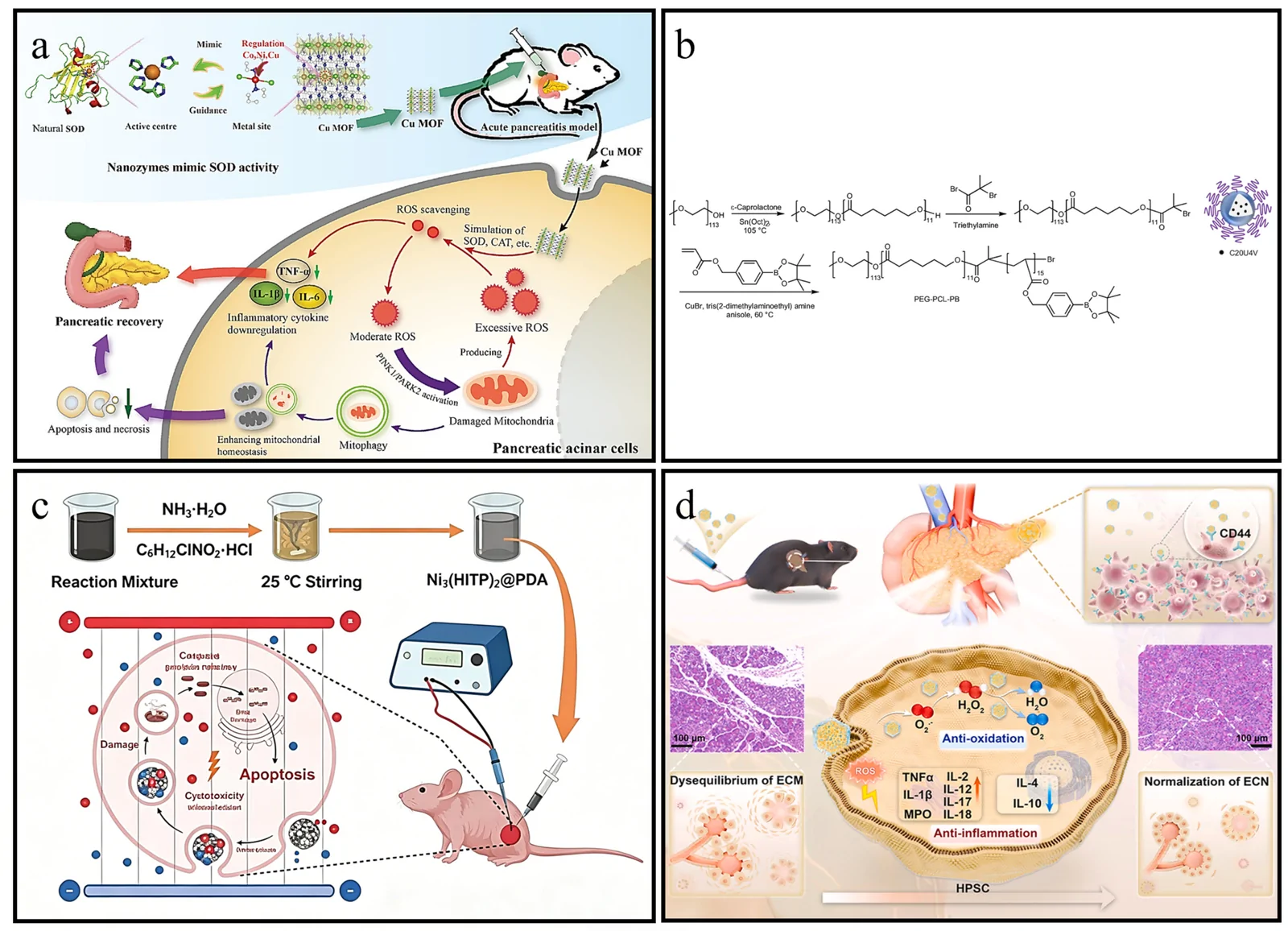
Representative MOF-based therapeutic platforms for pancreatitis and pancreatic cancer intervention. (**a**) Schematic illustration of the synthesis of Cu-MOF nanozymes and their application for reactive oxygen species scavenging and acute pancreatitis treatment [85]. (**b**) Preparation of the ROS-responsive nanoplatform M-C20U4V for disruption of iron homeostasis and enhancement of irreversible electroporation efficacy [86]. (**c**) Conductive MOF-assisted bipolar irreversible electroporation (IRE) system for improved electric field distribution and pancreatic tumor ablation [87]. (**d**) Targeted HC@CeMOF nanosystem for antioxidant and anti-inflammatory therapy of chronic pancreatitis through modulation of pancreatic fibrosis progression [88].

**Table 1:** Classification and engineering strategies of MOF-based platforms for pancreatic disease applications.

Classification Criteria	Representative Categories	Structural Characteristics	Influence on Biological Performance	Representative Applications in Pancreatic Diseases
Metal nodes	Zr-based MOFs (UiO series, PCN series)	High coordination stability and hydrolytic resistance	Improved structural integrity, prolonged circulation, and controlled degradation	Drug delivery, imaging, theranostics
	Fe-based MOFs	Redox-active metal centers capable of Fenton/Fenton-like reactions	ROS generation, ferroptosis induction, chemodynamic therapy, TME modulation	Ferroptosis therapy, stromal remodeling, ROS-mediated therapy
	Cu-based MOFs	Copper-mediated redox activity and Cu ion release	Cuproptosis induction, oxidative stress regulation, immune modulation	Cuproptosis-oriented therapy and immunotherapy
	Mn/Co-containing MOFs	Catalytic metal centers with enzyme-mimicking activity	Regulation of ROS balance and antioxidant/oxidative pathways	Nanozyme-based therapy and inflammatory disease intervention
Organic ligands	Porphyrin-based ligands (TCPP derivatives)	Extended π-conjugated structures and photoactive properties	Enhanced photodynamic/sonodynamic activity and imaging capability	Energy-triggered therapy and theranostics
	Phenolic ligands (tannic acid, gallic acid derivatives)	Metal–phenolic coordination networks	Antioxidant activity, ROS regulation, and bioactive functionality	Tumor microenvironment remodeling
	Functionalized ligands	Specific chemical groups for cargo interaction or responsiveness	Improved drug loading, targeting, and stimuli-responsive release	Targeted delivery systems
Pore architecture	Microporous MOFs	Uniform nanoscale cavities	Molecular-level cargo encapsulation and controlled diffusion	Small molecule drug delivery
	Mesoporous/hierarchical MOFs	Enlarged pore structures and interconnected channels	Enhanced loading capacity for macromolecules and combination cargos	Protein, nucleic acid, and combination therapy
Surface engineering	PEGylation, biomolecule modification, aptamer/peptide functionalization	Tunable surface chemistry	Improved stability, targeting ability, and biological compatibility	Tumor targeting and precision delivery
Biodegradability	Biodegradable metal–ligand coordination systems	Controlled framework decomposition under physiological or pathological conditions	Influences pharmacokinetics, clearance, and biosafety	Translational nanomedicine development
Stimuli responsiveness	pH-responsive, redox-responsive, ROS-responsive, magnetic-responsive systems	Dynamic structural transformation triggered by disease-associated signals	Controlled drug release and spatially selective therapy	PDAC therapy and theranostic platforms

**Table 2:** Representative MOF-based therapeutic and theranostic platforms for pancreatic diseases: mechanisms, advantages, limitations, and translational perspectives.

MOF Platform/Design Strategy	Therapeutic Cargo or Function	Mechanism of Action	Advantages	Current Limitations	Translational Status
Drug-loaded MOFs (UiO series, PCN series, ZIF-based systems)	Gemcitabine, paclitaxel, doxorubicin, SN-38	Improved drug encapsulation, controlled release, enhanced tumor accumulation	High loading capacity, protection of therapeutic molecules, reduced systemic toxicity	Limited clinical validation, scale-up challenges	Preclinical
Nucleic acid-delivery MOFs	siRNA, DNAzyme, gene-regulatory cargos	Gene silencing and regulation of tumor-associated pathways	Protection of nucleic acids and potential combination therapy	Biological stability, intracellular delivery efficiency	Early preclinical
MOF-based stromal remodeling systems	Collagenase, ferroptosis-inducing cargos	ECM degradation, CAF modulation, reduced collagen deposition, enhanced penetration	Addresses dense desmoplastic barrier of PDAC	Possible off-target ECM effects, incomplete stromal normalization	Preclinical
Fe-containing MOFs	Fe ions, ROS-generating cargos	Fenton chemistry, oxidative stress amplification, ferroptosis induction	Integrates catalytic therapy with tumor metabolism regulation	Metal ion toxicity and long-term fate require evaluation	Preclinical
Cu-containing MOFs	Copper ions and metabolic regulators	Copper homeostasis disruption and cuproptosis activation	Exploits metabolic vulnerability of PDAC	Copper accumulation and biosafety concerns	Early preclinical
MOF-based ROS-mediated platforms	Photosensitizers, sonosensitizers, catalytic centers	PDT/CDT/SDT/radiodynamic/catalytic nanozyme-mediated ROS generation	Spatiotemporal therapeutic activation	Hypoxia, energy penetration, heterogeneous response	Preclinical
Immunomodulatory MOFs	STING agonists, immune modulators, checkpoint-related cargos	Macrophage polarization, T-cell activation, immune microenvironment remodeling	Converts immunosuppressive TME into immune-responsive state	Complex immune interactions, limited clinical evidence	Preclinical
MOF-based imaging-guided theranostics	MRI/CT/fluorescence/PAI contrast components	Integrated diagnosis, localization, treatment monitoring	Enables real-time therapeutic guidance	Imaging standardization and regulatory approval remain challenges	Preclinical
MOF nanozymes for pancreatitis	Antioxidant catalytic systems	ROS scavenging, inflammatory regulation	Potential application beyond cancer therapy	Limited disease models and clinical evidence	Exploratory
MOF-assisted interventional platforms	Conductive MOF systems for IRE enhancement	Improved electrical distribution and local ablation efficiency	Expands MOF applications beyond systemic therapy	Device integration and clinical validation needed	Early translational stage

## Data Availability

No new data were created or analyzed in this study. Data sharing is not applicable to this article.

## Abbreviations

The following abbreviations are used in this manuscript:

MOFs	Metal–organic frameworks
PDAC	Pancreatic ductal adenocarcinoma
AP	Acute pancreatitis
CP	Chronic pancreatitis
ROS	Reactive oxygen species
TME	Tumor microenvironment
ECM	Extracellular matrix
PSCs	Pancreatic stellate cells
EMT	Epithelial–mesenchymal transition
GSH	Glutathione
GPX4	Glutathione peroxidase 4
TAMs	Tumor-associated macrophages
MDSCs	Myeloid-derived suppressor cells
CAFs	Cancer-associated fibroblasts
IDO1	Indoleamine 2,3-dioxygenase 1
STING	Stimulator of interferon genes
CDT	Chemodynamic therapy
PDT	Photodynamic therapy
SDT	Sonodynamic therapy
PAI	Photoacoustic imaging
MRI	Magnetic resonance imaging
CT	Computed tomography
PET	Positron emission tomography
CA19-9	Carbohydrate antigen 19-9
GMP	Good manufacturing practice
